# Operationalizing the Concept of Socio-environmental Tipping Points in River Basin Management

**DOI:** 10.1007/s00267-026-02585-z

**Published:** 2026-08-21

**Authors:** Julie Shortridge, Aleksi Räsänen, Hannu Marttila

**Affiliations:** 1 Department of Biological Systems Engineering, Virginia Tech, Blacksburg, VA USA; 2 Water, Energy and Environmental Engineering Research Unit, University of Oulu, Oulu, Finland; 3 Geography Research Unit, University of Oulu, Oulu, Finland

**Keywords:** Socio-ecological systems, Climate change, Land-use change, Land conservation and restoration

## Abstract

The concept of socio-environmental tipping points (SETPs) is gaining interest as a means to describe nonlinear transformations in socio-environmental systems that are driven by both biophysical and social dynamics. These SETPs stem from multiple interacting factors that can rarely be traced to a single driver, leading to significant complexity and uncertainty. Structured frameworks can support the rigorous identification and assessment of SETPs, but little research exists that applies them in practice. This research utilizes a previously developed conceptual framework to identify prospective SETPs, using the Kiiminkijoki River Basin in northern Finland as a case study location. Land use in rural, forested basins such as the Kiiminkijoki will play a crucial role in meeting Finland’s goals for climate change mitigation, water quality improvement, and biodiversity protection. However, they are facing many pressures, including legacy effects of peatland drainage, rapid climatic changes, and economic and demographic transitions. The conceptual framework was used to structure a 1-day workshop that brought together a transdisciplinary group of researchers and practitioners. Workshop participants used the framework to inventory the different social and environmental elements, processes, and linkages in the basin, and this inventory was then reviewed to identify prospective tipping points. The process resulted in five prospective SETPs where interactions between social and environmental processes create the potential for feedback loops and cascading changes. Our analysis forms a practical basis for improving the status of the Kiiminkijoki River, as well as a stepping stone towards applying the SETP framework to water management more broadly.

## Highlights

- Rapid socio-environmental change can occur through feedbacks and cascades
- Our structured process helps to characterize complex system interactions
- We applied the process to a Nordic river with land use and water quality challenges
- Example tipping points related to landowner perceptions and tourism development

## Introduction

Most environmental problems are driven by a combination of physical, biological, and social factors, especially in river basin systems. Recognizing these interactions, the concept of socio-ecological or socio-environmental systems (SES), where humans interact with the natural and built components of their environments (Elsawah et al. 2020), has long been used as a framing to support interdisciplinary understanding of complex environmental challenges (Berkes et al. 2000). In recent years, there has been increasing interest in the potential for rapid, systemic level changes in SES, often characterized as socio-environmental tipping points (SETPs) (e.g., Lenton 2020; Milkoreit et al. 2018). Multiple definitions of SETPs have been proposed, ranging from quasi-mathematical definitions based on amplifying feedback processes and bifurcation points (e.g., Lenton et al. 2022; Yi et al. 2025) to more general definitions centered on the idea of minor pressures or events that rapidly amplify into system-level changes (Tàbara et al. 2022; van Ginkel et al. 2020).

The tipping points concept has been applied to various SES issues, including primary industries such as fishing, forestry, and agriculture (Mathias et al. 2020; Yletyinen et al. 2019), clean energy transitions (Lenton et al. 2022; Winkelmann et al. 2022), climate change impacts and adaptation (van Ginkel et al. 2020; Fedele et al. 2020), water supply management (Berglund et al. 2023; Castilla-Rho et al. 2017; Krueger et al. 2019), land use change (Froese et al. 2023), and coastal and marine management (Cremin et al. 2024; Lauerburg et al. 2020; Shortridge et al. 2024). The concept is highly evocative, as it can describe potentially catastrophic social and ecological collapses (Harper et al. 2024), as well as more hopeful narratives of major, systemic transitions capable of preventing these collapses (Lenton 2020). However, this combination of broad applicability and emotional resonance creates a risk of overuse which could oversimplify the complex dynamics of natural and human systems (Kopp et al. 2025) and lead to unrealistic environmental policy ambitions (Milkoreit 2023). The concept has also been critiqued as being unsuitable for complex concepts like biodiversity which respond gradually and cumulatively to environmental stressors and are rarely governed by simple thresholds (Hillebrand et al. 2023).

Addressing these concerns requires empirical research that can elucidate SETP mechanisms and clearly demonstrate instances of their occurrence to advance understanding and inform management. However, prior research on SETPs has largely been conceptual or theoretical (Lauerburg et al. 2020; Yletyinen et al. 2019). One challenge in moving from conceptual to empirical research on SETPs is the presence of significant complexity and uncertainty, as SETPs likely stem from multiple drivers, occur across multiple pathways, and can lead to multiple post-tipping states that are difficult to predict (Cremin et al. 2024; Mathias et al. 2020; Winkelmann et al. 2022). This complexity presents a significant challenge to the empirical study of SETPs, with some arguing that new methodological and computational modeling approaches are needed to observe tipping dynamics within SES (Juhola et al. 2022; Milkoreit 2023). Conceptual frameworks can support empirical research into SETPs by providing a means for organizing complex phenomena and questions in a structured way. We refer to a framework as a basic vocabulary of concepts and terms that can be used to organize inquiry and develop causal explanations for system behavior (McGinnis and Ostrom 2014; Ostrom 2005). This is particularly important for problems that require collaboration across disciplinary boundaries to learn and take action in coordinated way (Harper et al. 2024). Conceptual frameworks are also important in determining the requirements of new analytical or modeling approaches that can deal with the complexity of the system in question (Currie et al. 2023).

Multiple conceptual frameworks have been proposed as a means for organizing information on SESs generally and SETPs specifically. A classic SES framework has an emphasis on broad tiers of governance and resource systems and a focus on “action situations” as a driving force for system change (McGinnis and Ostrom 2014; Ostrom 2009). Other frameworks outline the relationships between political, technological and behavioral changes (Fesenfeld et al. 2022), identify sequential processes of human action that can lead to desirable SETPs (Moore et al. 2014), and center the social dimensions of desirability and intentionality (Graham et al. 2023). These approaches provide a thorough accounting of the different social institutions and processes that influence environmental change, as well as the potential for human actions to encourage tipping points. Some other frameworks place a greater emphasis on physical and technological changes that can be represented quantitatively, using concepts such as cross-impact matrices, critical control values, and system monitoring to characterize abrupt changes (Froese et al. 2023; Lenton et al. 2022; van Ginkel et al. 2020). However, these approaches provide little guidance on how these critical components (crucial system features, control parameters, critical values, enabling conditions, etc.) can be empirically identified, characterized, or quantified. More recent work aims to support this need by organizing physical and social system features using a common set of terminology and concepts that describe system’s characteristics and functions as a whole (Shortridge et al. 2024). While this conceptual framework has been reviewed by a panel of researchers and practitioners involved in coastal SES management (Shortridge et al. 2024), it has not yet been applied to a real-world case study in river basin management.

The objective of this paper is to demonstrate how this conceptual framework (Shortridge et al. 2024) could be operationalized to identify prospective SETPs, as well as data collection priorities that would support their empirical study, using the Kiiminkijoki River Basin in Northern Finland as a case study. This case study also provides a novel application of the SETP concept, as it is the first time it has been applied to boreal conditions, in a basin with heterogenous land use, land ownership, and several interacting environmental challenges. The Kiiminkijoki is an ideal case study location as many of the issues it is facing are reflective of issues facing the Nordic region more broadly. We applied the conceptual framework using a semi-structured expert elicitation approach during a transdisciplinary researcher-practitioner workshop. Workshop participants were first guided through the process of developing an SES inventory relevant to land use and water quality. They then reviewed this inventory to identify and characterize prospective SETPs that would result in significant, rapid changes in socio-environmental conditions in the basin. We demonstrate how the conceptual framework was used to support structured transdisciplinary inquiry into SES tipping points in the Kiiminkijoki River Basin and describe the SES tipping points identified through this process.

## Methods

### Overview of Conceptual Framework

For the purposes of this work, we define tipping points as: “*cases when a small perturbation or change, either due to external forcing, individual activities, or targeted intervention, leads to a qualitative change in the future state or trajectory of a complex system.”* This definition is an integration of those proposed by Lenton (2020 and 2022) and (Tàbara et al. 2022) and allows for the possibility of both desirable and undesirable tipping points, as well as allowing for them to be intentionally and unintentionally induced. Thus, this definition aligns more closely with the concept of tipping points being minor pressures or events that amplify into system-level changes (van Ginkel et al. 2020), rather than those focused on feedback processes specifically (e.g., Yi et al. 2025).

**Fig. 1:**
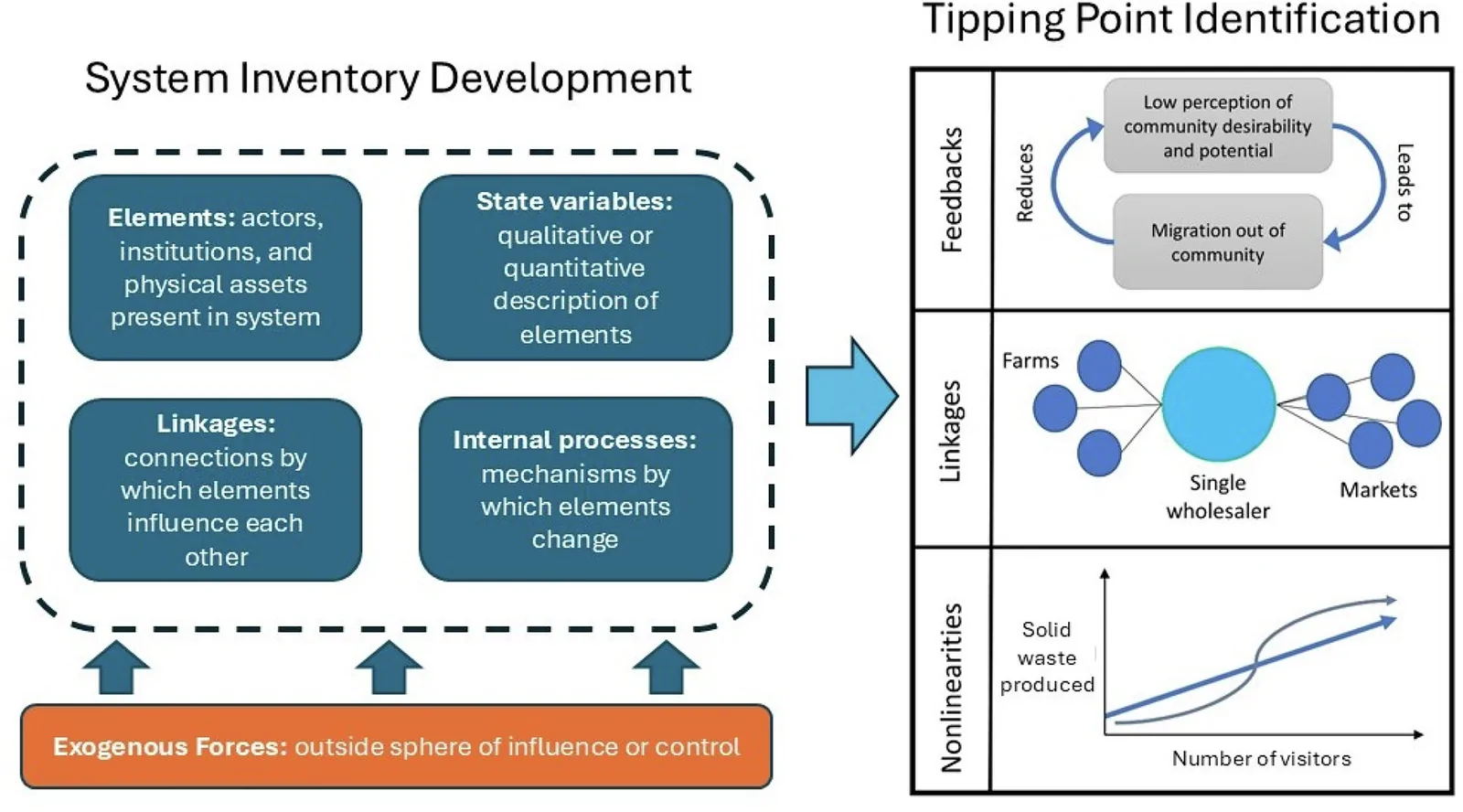
Schematic of implementation process of the conceptual framework

The conceptual framework by Shortridge et al. (2024) first develops an inventory of the different components, relationships, and processes of change within an SES, and then evaluates how the SES components and structures could contribute to SETPs (Fig. 1). The system inventory is structured around five components (Table 1). The first three components describe the system at a single point in time by identifying the *elements* that exist within the system, the *state variables* that describe those elements, and the *linkages* that describe how different elements interact and influence each other. To incorporate descriptions of change, system dynamics are organized by distinguishing *internal processes* which can be meaningfully influenced by systems actors and *exogenous forces* which cannot, while recognizing that this division is subjective and that many processes may be partially influenced. After the SES inventory has been developed, it is reviewed for three potential tipping mechanisms. *Feedback processes* occur when the state variables that contribute to a process are impacted by the same change in either stabilizing or amplifying feedback loops. *Cascading linkages* occur when the structure of linkages across SES elements create conditions where marginal changes in the state variable of one element impacts multiple additional elements. *Nonlinear relationships* between state variables can lead to threshold points where a slight change in one state variable causes a large change in another. These different mechanisms are not necessarily exclusive to each other, and in many examples could occur simultaneously. More discussion on the rationale and specifics of this conceptual framework is presented in the supplementary material.

**Table 1:** Summary of conceptual framework components

System inventory components		
Name	Description	Examples
Elements	The actors, institutions, and physical assets that are present in the system of interest and influence its resilience and sustainability.	Residents, government agencies, community organizations, water bodies, farms, protected land.
State variables	Quantitative or qualitative descriptions of the elements in an SES in terms of physical, institutional, economic, and socio-cultural characteristics.	Population, values, goals, financial resources, water quality, land use
Linkages	Connections between elements within the SES where a change in the state variable of one element impacts the state variable of another.	Physical connections, financial interactions, cultural influences, regulatory authority
Internal processes	Mechanisms by which the elements and state variables change over time; these processes can potentially be influenced by actors within the SES.	In- and out- migration, restoration, construction of protective measures, changes to land management practices
Exogenous forces	Factors that influence the elements of the system and cannot be meaningfully influenced by actors within the SES.	Climatic change, extreme weather events, EU and national-level regulations
Tipping mechanisms		
Feedback processes	The state variables that contribute to a process are impacted by the same process.	Out-migration from a community leading to lower perceptions of community desirability.
Cascading linkages	The structure of linkages within an SES allows changes in one element to propagate out to many others through central elements or chains of linkages.	Loss or creation of an institution that connects many different elements; domino effects.
Nonlinear relationships	Conditions where a slight change in one state variable causes a large change in a linked variable.	Water temperature thresholds that impact species survival.
Combination	Conditions where multiple tipping mechanisms occur simultaneously.	Feedback processes that rely on linkages across multiple elements; nonlinear relationships that impact multiple elements through linkages.

### Study Area

**Fig. 2:**
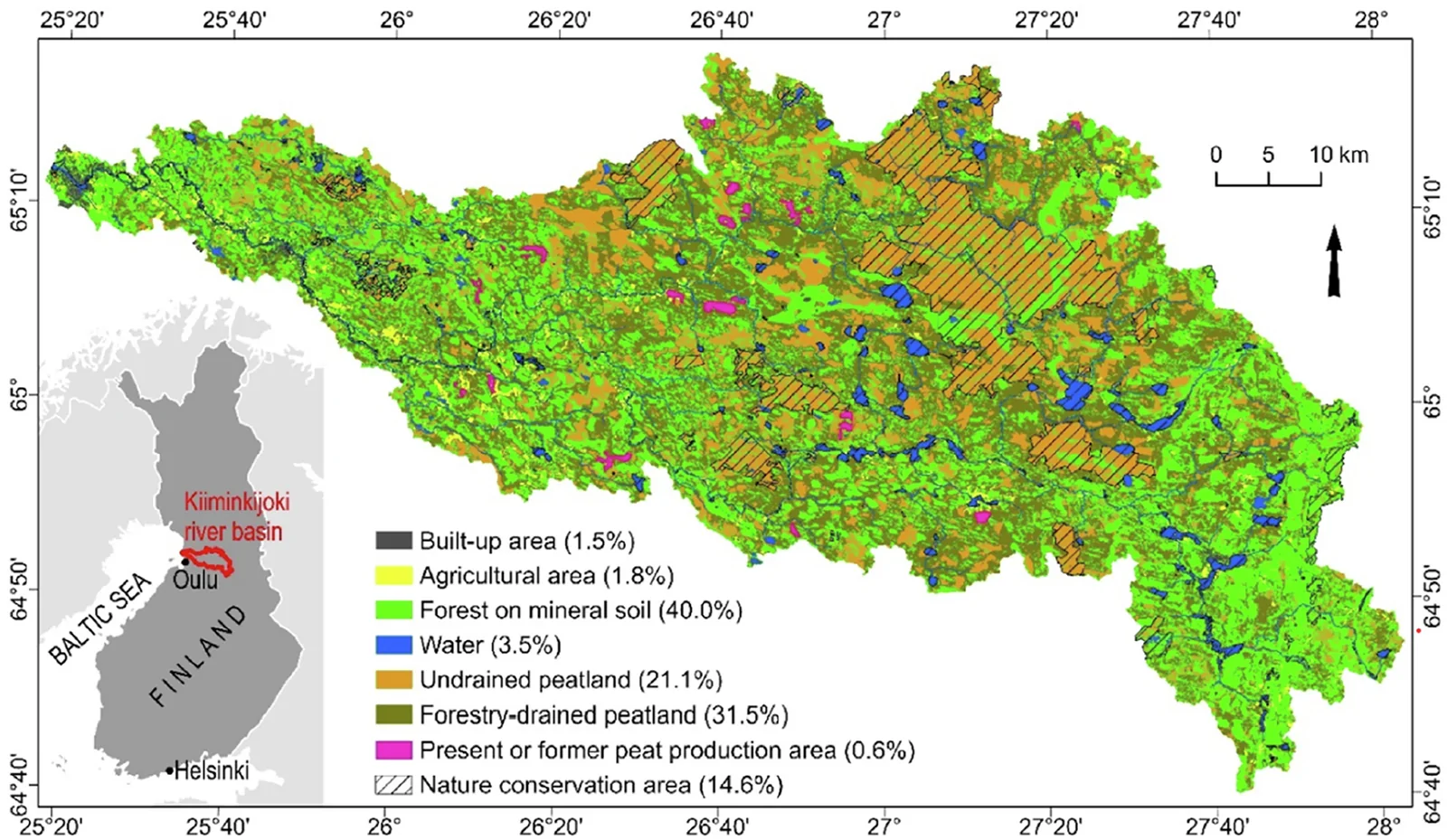
Location and land use of the Kiiminkijoki River Basin. From Sarkki et al. (2024), Creative Commons 4.0 International License

The Kiiminkijoki River Basin is a rural area covering 3824 km^2^ in northern Finland (Fig. 2). Approximately half of the basin area are comprised of peatlands, but 60% of these have been drained in the past century to support forestry, agriculture, and peat extraction. Many drained peatland forests in the Kiiminkijoki basin and Finland more broadly are unproductive due to nutrient deficiencies, and some are no longer economically profitable (Juutinen et al. 2020; Ecological Economics). A large portion of the basin is under state ownership by the Metsähallitus (the Finnish state forest administration) which manages lands for both conservation and commercial forestry, and 58% of the basin is privately owned by mostly small landowners. The basin is located across four municipalities within two regional administrative areas, creating challenging conditions for shared governance. Approximately 15% of the basin is protected by various conservation programs. Water quality in the river system ranges from poor in some tributaries to excellent in some of its uppermost reaches. Water quality impacts mainly stem from nonpoint source introduction of nutrients and organic matter from peatland drainage and forestry operations (Nieminen et al. 2025). This has contributed to the disappearance of salmon and other migratory fish, which are an important part of local culture and pride (Sarkki et al. 2024).

The Kiiminkijoki basin was the focus of a recently completed project, “Co-planning of land use sector climate change mitigation in the Kiiminkijoki river basin (MATKI)” conducted by National Resources Institute Finland, University of Oulu, and Finnish Forest Centre. The transdisciplinary project developed a land use road map and future vision for the river basin, with its key methods including stakeholder workshops, key informant interviews, and environmental modeling. A critical conclusion from the project was that to make true changes in the basin, implementation of land use measures does not suffice; instead, larger changes in actor collaboration and wider society are required (Räsänen et al. 2024).

### Workshop Details

We convened a 1-day transdisciplinary researcher-practitioner workshop in January 2025 to collaboratively identify and characterize SETPs for the Kiiminkijoki Basin. The workshop was attended by 17 researchers and practitioners from Finnish universities, government research institutes, and regional and municipal administrative offices. The researchers had a background in a range of disciplines, including hydrology, ecology, anthropology, geography, and engineering, while the practitioners had a particular focus on environmental and basin management. Participants were identified from the authors’ professional networks and from recommendations of other researchers involved in the study and management of the basin. We recognize that drawing participants from this small network likely presents a limitation, and future efforts could benefit from broader recruitment efforts. In particular, representation from key actors within the basin, such as private and institutional landowners, would be an important step in incorporating local knowledge and encouraging ownership over the process.

Prior to the start of the workshop, we developed a baseline SES inventory of the basin using the conceptual framework developed by Shortridge et al. (2024). This baseline inventory served as a starting point for workshop discussions and provided examples of the different components of the conceptual framework. At the beginning of the workshop, we briefly presented an overview of the conceptual framework and how it would be used to identify prospective SETPs (see Supplementary Material section S.5). We then led the workshop participants through a series of small group discussions aimed at identifying elements, state variables, linkages, processes, and exogenous forces related to land use and water quality in the basin. The small groups consisted of four to six participants and were determined ahead of the workshop to ensure that each group included a balance of disciplinary expertise. Small group discussions were organized to last for 20–30 min and were generally followed by full-group report-outs for synthesizing discussion. After this inventory had been developed, we conducted a second series of small group discussions aimed at identifying and describing already occurred and potential future SETPs based on the mechanisms of feedback processes, cascading linkages, and nonlinear relationships. At each stage of the workshop, participants worked in small groups and recorded the outcomes of their discussions on printed or electronic worksheets that mirror the structure of the tables and figures presented in this manuscript. During the small group discussions, the facilitator circled the room to check in with participants and be available to answer questions. However, they only offered guidance or assistance when asked, so that the participants’ outputs would not be overly influenced by the facilitator’s perspective.

After the workshop concluded, the results from individual groups were combined into a final system inventory and SETP description by the authors. This entailed combining ideas, as different groups came up with system inventory components and prospective SETPs that were different from each other but not contradictory. Nothing was removed from the final system inventory, but author discretion was used in determining which aspects were presented in the manuscript tables and which were only in the supplementary material. We prioritized the inclusion of inventory components that generated discussion and related to the identified tipping points. Additionally, the manuscript authors made informal observations during the process and in response to the outputs produced. While these observations were not collected as formal data, they did inform the discussion and interpretation of results.

## Results

### System Inventory Outcomes

Landowners are a key element that was broken down into private and institutional landowners, who are likely to have very different objectives, perceptions, and resources available to implement improved practices. This means that different state variables are needed to describe private versus institutional landowners (Table 2 and supplementary material). For instance, the age structure of private landowners was considered very important because many landowners are retiring without clear future plans for their property. As younger generations move to more urban areas, this has created many remote landowners who have inherited lands but are not closely involved in their day-to-day management. Institutional landowners include the Metsähallitus that manages owned by the Finnish state, as well as holdings by municipalities and parishes. While state variables that describe operations, such as land area and management practices, may be the same for institutional and private landowners, other variables that describe the motivations and options available to these landowners are different. It should be noted that elements are not necessarily mutually exclusive of each other, and many elements may exhibit considerable overlap. For example, many (but not all) private landowners may also have farms or production forests on their property and these individuals may be active in advocacy and community groups as well.

**Table 2:** Selected elements and descriptive state variables within the Kiiminkijoki SES

Elements	Descriptive state variables
Private landowners	• Population: size, age structure, residence (local or remote) • Operations: area of land operated, income (sole or partial income from land), management practices in use, financial and technical resources • Utilization of services • Level of participation in community efforts • Values (relative value of environment, economy, etc.) • Perceptions (e.g., perceived feasibility of new technologies)
Municipal governments and regional administrations	• Mandate and responsibilities • Goals • Resources (financial, expertise, etc.) • Participation in regional and national initiatives
Institutional landowners	• Operations: area of land operated, management practices in use, income, role of farming/forestry in income • Resources (financial, staff, etc.) • Mandate, responsibilities, and goals • Policy constraints • Level of participation in community efforts, services provided to community
Advisory and advocacy organizations	• Goals • Financial and technical resources • Number of participants • Participation in community efforts
Farms and production forests	• Operations: size, income (including subsidies or participation in carbon and biodiversity markets), activities (e.g., livestock, forestry, crops, reindeer herding), management practices in place, financial and technical resources • Employees and labor available • Environmental conditions: rate of nutrient loading and GHG emissions, drainage system in place, conservation or protection status, soil conditions (mineral or peatland)
River, streams, lakes	• Pollutant and nutrient concentrations • Flow rate regime, including seasonal variations • Conservation or protection status
Restored areas	• Areal extent • Restoration conditions and characteristics • Rate of nutrient loading or retention
Species	• Population • Health (disease levels, reproduction rates, etc.) • Native versus invasive

The complete list and description are included in the Supplementary Material

**Fig. 3:**
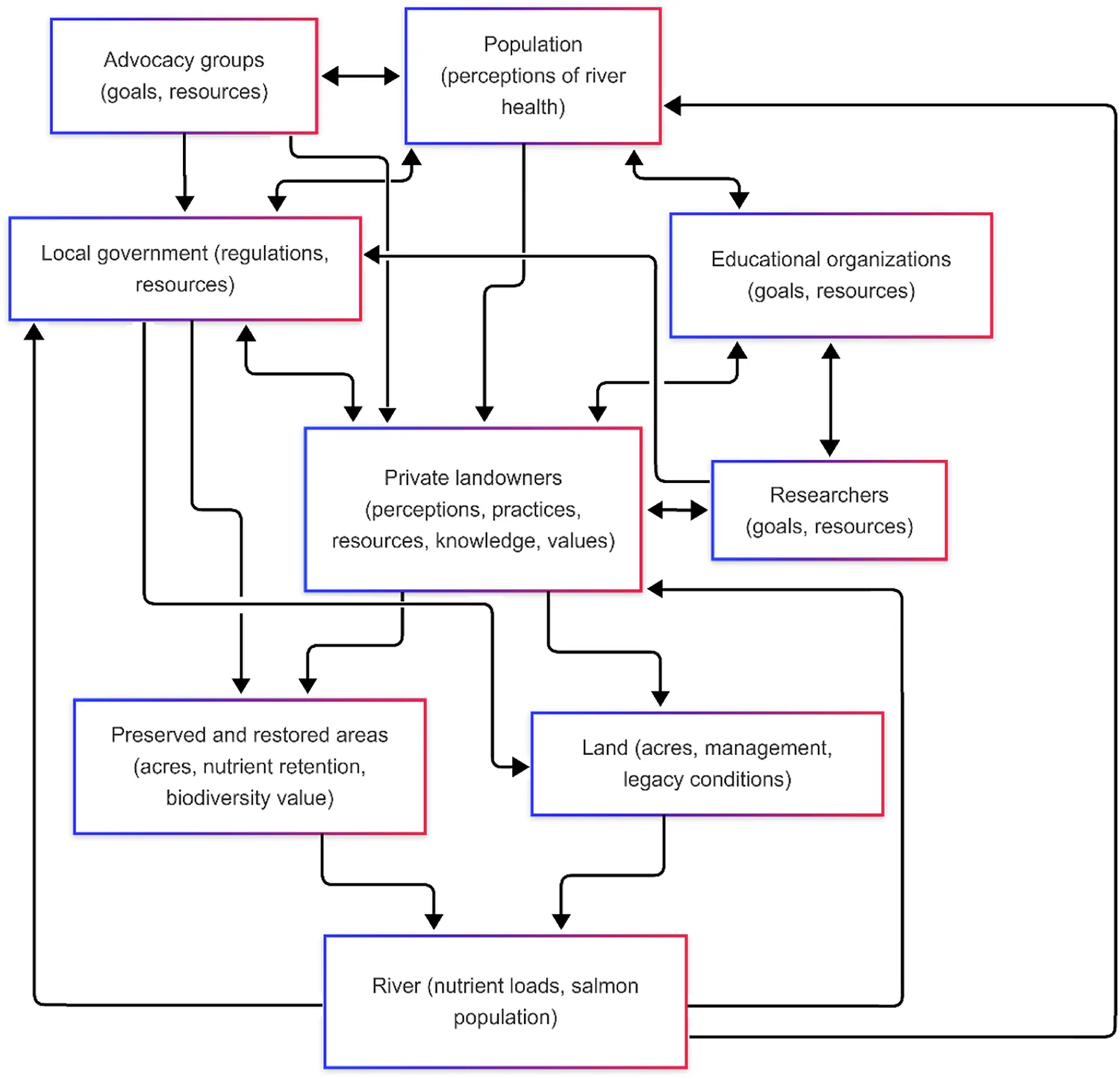
A simplified example of linkages between elements (in boxes) and their associated state variables (in parentheses) relevant to land use and nutrient loading the Kiiminkijoki basin

Relevant linkages between elements and state variables were specifically centered on private ownership land use and nutrient loading in the river (Fig. 3). Key actors who influence land use and restoration (private landowners) are influenced by multiple social and physical elements. For example, educational organizations and local government requirements can both influence landowner perceptions, knowledge, and values, which in turn will influence the actions that they take. Observable variables related to river water quality (such as turbidity, water color, or fish population) will influence the perception of river health in landowners as well as the population more broadly. While mapping an exhaustive account of all linkages in an SES is likely to be unpractical, even a subset of linkages most relevant to the process or element of main concern can highlight connections and influences across physical and social components of the system.

Processes describe mechanisms by which the system can change over time, as well as the elements and state variables that drive the process and are impacted by it (Table 3). For example, the construction of restoration measures will be influenced by a range of physical and social factors. This includes the land itself, the income it provides to landowners, as well as the availability and resources of supporting industries. In many cases, the elements that drive a process are impacted by that same process, creating the potential for feedback loops.

**Table 3:** Selected processes relevant to land use change and water quality within the Kiiminkijoki SES

Process name	Process drivers		Process impacts	
	Elements	State variables	Elements	State variables
Application of restoration and protection measures	Farmed and forested land	Productivity levels, soil conditions (mineral or peatland)	Restored and protected land	Nutrient retention, carbon and greenhouse gas balance, biodiversity
	Forest and farm landowners (private)	Current income from farming and forestry, financial resources, technical understanding of options, perceptions of restoration benefits and feasibility	Forests and farm landowners (private)	Perceptions of water quality and biodiversity, perceptions of benefits and feasibility of restoration and protection projects
	Educational and advisory organizations	Resources, goals, number of participants		
	Related industries (e.g., construction, materials)	Income, financial and technical resources, labor available		
Farming and forestry management changes, including formal land conservation	Farmed and forested land	Productivity levels, soil conditions (mineral or peatland)	Farm and forested land	Nutrient retention, carbon and GHG balance, biodiversity
	Farm and forestry landowners (private)	Income from farming and forestry, financial resources, technical understanding of options, perceptions of benefits and feasibility, values	Farm and forestry landowners (private)	Income from farming and forestry, technical understanding of options, perceptions of benefits and feasibility
	Localities and regional administration	Constraining or encouraging regulations, presence and resources available for incentive programs	Agricultural and forestry related businesses and industries	Income, perceptions of value
	Researchers	Resources, goals, funding, projects		
	Educational and advisory organizations	Resources, goals, number of participants		

The complete list and description are included in the Supplementary Material

The final components of the system inventory are exogenous forces (Table 4). These are similar to processes in that they are a mechanism for change within the basin but cannot be meaningfully influenced by actors and elements within the system. For example, rising temperatures and changing hydrology caused by changing climate will influence physical conditions in the basin (such as nutrient loading rates and forest growth), as well as management practices as landowners respond to changing climate conditions. While forestry, agricultural, and peatland management practices in the basin can influence carbon sequestration and storage and thus mitigate climate change, the effect is small relative to emissions on a global scale. In the context of regulatory or economic incentive programs, the distinction between exogenous forces and internal processes often depend on the scale over which they are implemented. In the Kiiminkijoki basin, the most relevant programs are occurring at the national or EU levels, but this would not necessarily be the case in other contexts.

**Table 4:** Selected exogenous forces with the potential to impact land use change and water quality within the Kiiminkijoki SES

Exogenous Force	Elements acted upon (state variables in parentheses)
Warming temperatures	Land (nutrient form, vegetation growth), landowners (management practices), river (species, water quality), biodiversity, pests and invasive species
Hydrologic changes (e.g., heavier rainfall; winter shortening, longer dry periods)	Land (vegetation growth, nutrient losses), landowners (management practices), river (turbidity, water quality), groundwater levels (higher acid sulfite when lower due to drought)
EU and national level regulations (e.g., restoration law, water directive)	Local governments (resources, mandate), landowners (management practices)
Market demand for products, including green energy and bioeconomy products	Farmers and forest-owners (income, management practices), land (ownership)
EU and national level carbon and biodiversity markets (development, demand)	Landowners (perspectives, incomes, and values, management practices)
Migration patterns and demographic change in other locations	Residents, landowners, houses, land, local businesses and services
Social attitudes (nationally and regionally) and awareness of conservation policies	Peatlands (through regulation and forest economics), forests (through regulations and economics), agricultural lands (management), land use patterns more broadly, land ownership, municipalities, businesses

### Identified Socio-Environmental Tipping Points

After the system inventory for the Kiiminkijoki basin was developed, workshop participants tasked with identifying and describing basin SETPs stemming from the system inventory, which could encompass previous SETPs that had already occurred in the basin, as well as prospective SETPs which could potentially occur in the future. Initial SETPs were identified and described in small groups and then discussed among the full group of participants. Workshop participants identified and described five key SETPs that were both feasible (i.e., participants felt that there was a reasonably high likelihood) and impactful (Table 5). The tipping point descriptions below provide a synthesis of the workshop discussions as well as author perspectives on how these discussions related to broader issues and research within the SES literature.

**Table 5:** Summary of socio-environmental tipping points (SETPs) related to land use management and change identified for the Kiiminkijoki River basin

SETP name	Description	Tipping mechanisms	Key elements involved (state variables in parentheses)
Improved perceptions of restoration projects	Successful restoration projects lead to observable benefits (e.g., better water quality, increased land values), resulting in greater perceived feasibility and benefits.	Cascading linkages, feedback processes	Restored peatlands (area, esthetic quality), river system (water quality, salmon population), residents and private landowners (perceptions of river and land health, perceptions of value and feasibility of restoration), land areas (economic value from restoration and carbon market laws)
Sustainable tourism growth	Nationally recognized biodiversity hotspot status prompts increased tourism, the revenues of which are used to fund additional restoration projects.	Feedback processes, cascading linkages	Metsähallitus (resources, aims, and activities), land (esthetic quality, biodiversity, conservation status), river (water quality, fish population), tourism companies (quality of services, resources, prices, revenue), visitors (demand for visits, perceptions of area), local government and businesses (revenue, resources for conservation)
Demographic and livelihood changes	Demographic changes (e.g., aging population or growth in remote workers from urban areas) change local values to increase or decrease interest in restoration.	Cascading linkages, feedback processes	Residents (age, values), infrastructure (condition), local governments (service provision, resources), companies (job opportunities, remote work policies, local shops and services), land (demand for housing and recreation, percent local ownership)
Basin Coordinator Position	Establishment of a river basin coordinator creates a hub connecting many other elements	Cascading linkages	Institutional landowners (resources, mandates, participation in community efforts), private landowners (resources, utilization of services, perceptions), municipal governments and regional administrations (resources, goals, participation in regional initiatives), research organizations (goals, current projects); Communication and strength of interactions across all elements
Environmental thresholds	Many relationships between environmental elements are nonlinear with thresholds beyond which species’ survival drops significantly.	Nonlinear relationships	River (temperature, pollutant and nutrient concentrations), species (population, disease levels, reproduction rates)

#### Feedback Loops related to Land Management and Restoration Perceptions

**Fig. 4:**
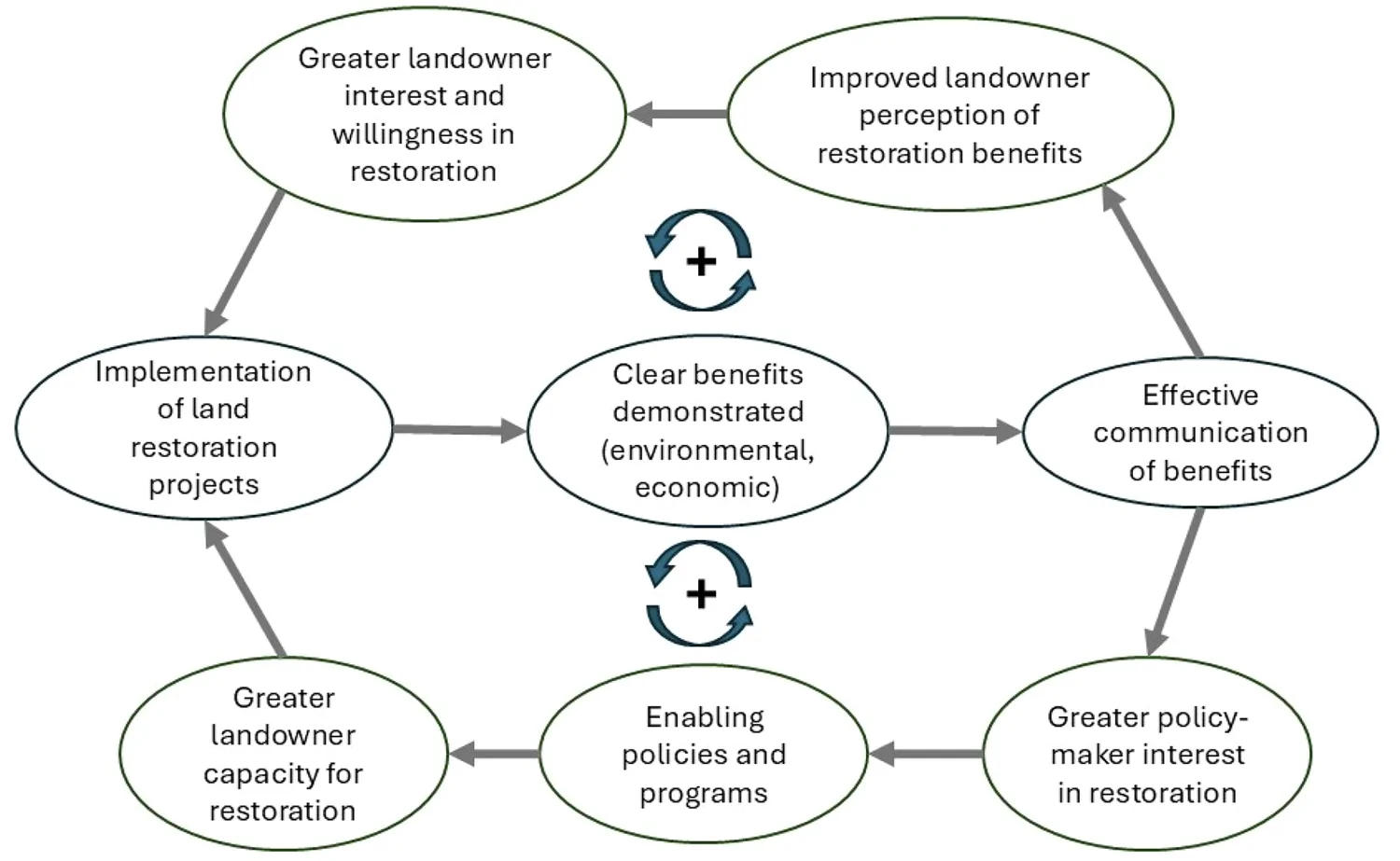
Feedback processes involved in the land management and restoration perceptions tipping point

Because relatively small land parcels under private ownership are common in the Kiiminkijoki basin, any effort at improving environmental conditions will require landowner buy-in. This SETP relies on feedback loops (Fig. 4) that could be strengthened by using successful examples of restoration projects to improve landowner perceptions of the feasibility and benefits of restoration actions. By encouraging enabling policies and programs, feedback loops could also create greater landowner financial and technical capacity for implementing restoration projects and improved management practices. This tipping point aligns with the concept of “proximising” benefits of nature restoration to impact the value placed on natural ecosystems (Gittins et al. 2024). Landowner perceptions are often quite different than those of researchers or governmental institutions. For example, scientific knowledge on continuous cover forestry suggests that it can have many environmental benefits, but stakeholders in the Kiiminkijoki saw this practice as infeasible in most conditions (Räsänen et al. 2024). Prior workshops in the basin suggest that stakeholder values related to land management are not necessarily in conflict with each other, and a strong majority of participants (82%) were highly motivated to improve water quality in the river. However, most disagreement stemmed from perceptions about which land management measures would be most effective in achieving this shared vision (Sarkki et al. 2024), a situation that has been observed in other peatland regions as well (Lees et al. 2023).

Many factors influence landowner perceptions, creating different avenues for encouraging feedbacks between successful implementation of restoration projects and landowner perceptions of them. Previous research has demonstrated that interest in agricultural land restoration tends to be highest among landowners who previously engaged in restoration efforts (Jellinek et al. 2013), suggesting the potential for reinforcing feedback loops that begin with small restoration projects. Because interpersonal relationships are also highly influential on landowner perceptions of restoration programs (Rosenberg 2007), peer-to-peer sharing among landowners could also be an effective means to encourage interest in restoration. Different actors can support different approaches to encourage replication. For example, scaling down (ensuring the means for policy to support adoption) requires policy actors and better information from research organizations, while scaling out (replicating practices) requires platforms and mechanisms for sharing information across different locations (Sarkki et al. 2024). However, there are also challenges that can create a disconnect between restoration success and landowner perceptions. For example, achieving measurable benefits at the basin scale is a key concern expressed in prior workshops (Räsänen et al. 2024), especially when even successful restoration may take years before their benefits are fully apparent. Researchers can play a role in addressing this challenge by developing monitoring and modeling programs that capture environmental improvements from restoration before those benefits are easily visible.

#### Nature-based Tourism Development

**Fig. 5:**
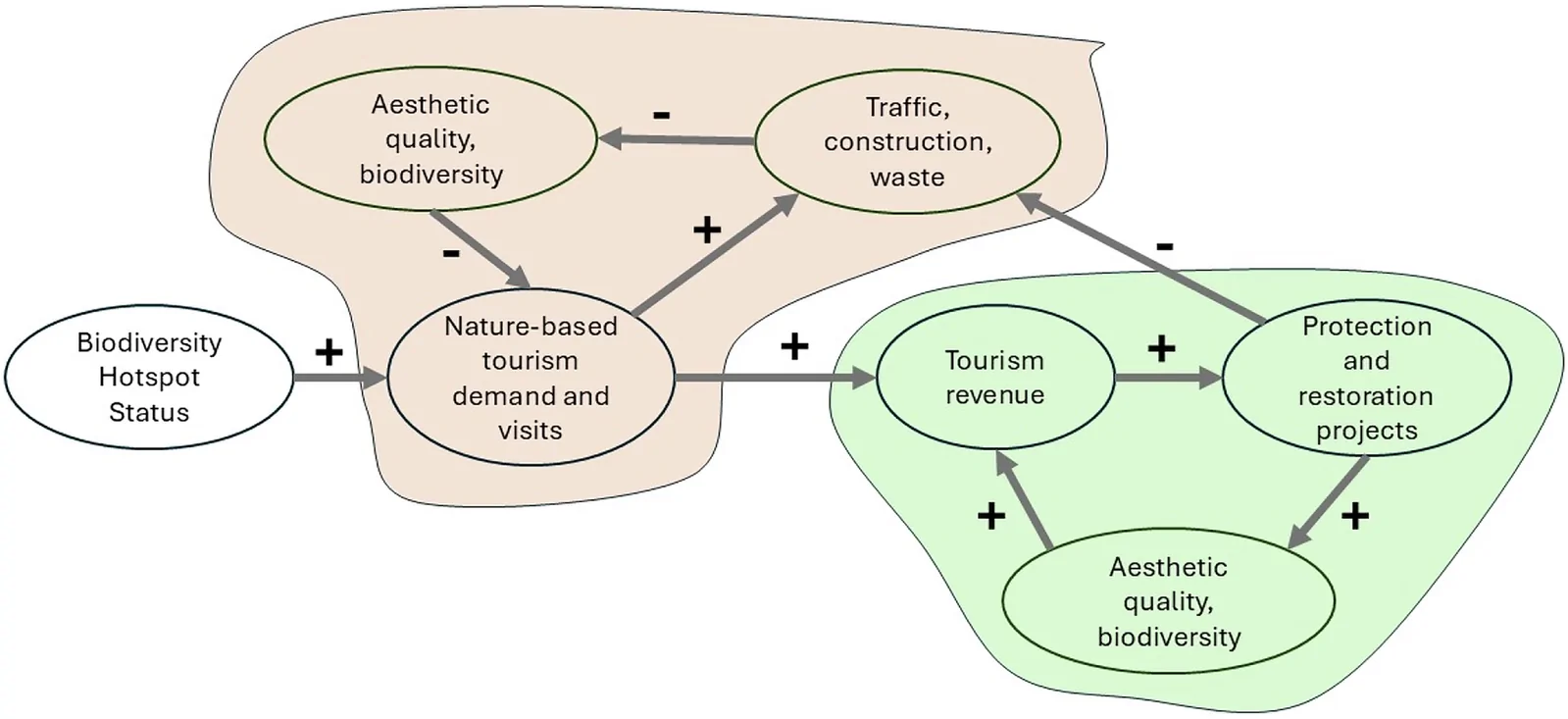
Nature-based tourism feedback loops stemming from the potential designation of the Kiiminkijoki basin as a biodiversity hotspot. Plus and minus signs indicate positive and negative relationships. The red-highlighted loop presents negative feedback which could lead to a degradation of environmental conditions in the basin, whereas the green-highlighted loop presents a positive feedback loop where increased tourism revenue provides an ongoing funding stream for land protection and restoration projects

A second potential SETP was growth in nature-based tourism. The Kiiminkijoki is currently under consideration to be designated a nationally-recognized biodiversity hotspot where nature restoration and management measures will be prioritized. While the immediate impact of this designation would be an influx of resources to support conservation, participants expected that the scale and high-profile nature of the designation could also be used to increase interest in nature-based tourism to the area. This could create different forms of feedback loops that either support or hinder conservation, creating potential for both desirable and undesirable feedback loops (Fig. 5). Additional visitors would lead to increased revenue from tourism, but also potentially more traffic, construction, erosion, and other environmental disturbances. If these disturbances are significant enough to reduce the esthetic quality of the landscape or opportunities for wildlife viewing, this could dampen tourism interest in the area. This creates a negative feedback loop (highlighted in red in Fig. 5) that would push the system back towards its current state with limited tourism and a degraded environment. However, participants also envisioned an alternative pathway where additional revenues would be funneled towards the implementation of additional restoration projects. Payment for ecosystem services generally, and landscape and recreational values more specifically, could serve as one mechanism for doing this and has been applied in other areas of Northern Finland (Mäntymaa et al. 2018). This could potentially create a lasting funding stream for nature-based solutions in the basin and lead to a positive feedback loop (in green) where better environmental conditions are leveraged into increasing tourism revenues.

Workshop participants’ perceptions that tourism growth in the basin could progress in different ways is consistent with research about the relationship between nature-based tourism and environmental conservation more broadly. Prior research has demonstrated how different approaches to visitor access, environmental education, and communication can lead to very different environmental outcomes in tourism areas (He et al. 2023; Wolf et al. 2019). Studies in Finland have shown that tourists do have a willingness to pay for biodiversity and landscape quality (Tyrväinen et al. 2014), but that effective financial means for tourism to support nature protection is a key area of needed research (Fredman and Margaryan 2021). A complicating factor is Finland’s history of “Everyone’s rights,” which allow people to freely access natural areas (including private property) for low-impact activities such as hiking, fishing, and berry picking. There are concerns that increasing use of wilderness sites could lead to their deterioration (Ervola et al. 2024), and additional research is needed to help decouple tourism growth in the basin from environmental harm.

#### Demographic and Livelihood Changes

A third potential SETP was changes in demographic characteristics and feasible livelihood models in the basin, many of which have the potential to create feedback loops in different directions. Many of the small villages and towns in the Kiiminkijoki basin have been experiencing population loss, especially of younger residents. This leads to many challenges, including the loss of public services and facilities such as schools and healthcare centers, fewer financial resources for infrastructure development and maintenance, and fewer economic opportunities for residents. This has also created a common situation of remote land ownership when younger generations living in urban centers inherit their family’s land but do not reside on it. The demographic processes driving population decline contain multiple positive and negative feedbacks (Alados et al. 2014) but are complicated by the rise in remote work arrangements following the COVID-19 pandemic (Bogason et al. 2024).

These demographic trends can intersect with land use in several ways that create the potential for socio-environmental feedback loops. For example, prior research has indicated that interest in land restoration programs is highest among landowners who earn most of their income off-land, whereas those who are more economically reliant on land tend to be more motivated by short-term productivity and profitability (Jellinek et al. 2013). Thus, it is possible that a growth in landowners who are not financially reliant on forestry income would lead to greater adoption of restoration measures. More broadly, these demographic changes can influence local values and norms, entrepreneurship and social innovation, but also create tensions between newcomers and long-term residents (Álvarez-Montoya and Ruiz-Ballesteros 2024). Given the importance of local connectivity and cohesion in supporting quality of life in rural areas (Peters, 2019), it is important that rural development programs encourage local participation and trust building rather than economic growth alone (Dax and Fischer 2018).

#### Establishment of Basin Coordinator Position

The fourth SETP was the establishment of a basin coordinator. The basin is located across four different municipalities and includes both state- and privately-owned lands, with numerous stakeholders involved in land management. Governance of the basin must account for multiple environmental and social targets, including water quality, biodiversity, carbon sequestration, and the needs for local livelihood opportunities. Additionally, the need for new administrative and governance structures to better support cooperation and interaction was recently concluded in Kiiminkijoki river basin (Räsänen et al. 2024). This led to the recommendation and creation of a basin coordinator position to coordinate restoration measures, support the co-production of knowledge, and facilitate interaction and cooperation with various stakeholders. In January 2025, a new project was launched in which the Finnish Forest Centre (Metsäkeskus) started to develop a basin coordination structure for the area.

While the establishment of this position is not necessarily an SETP in and of itself, workshop participants considered it an important step that could encourage positive tipping in the future. The key reason for this is that the establishment of the basin coordinator position restructures linkages within the system by acting as a hub that facilitates the sharing of information between different actors and institutions across the basin. This can encourage tipping by strengthening linkages and creating potential for cascading change. Additional links between system actors and institutions can also encourage feedback loops, for example by connecting landowners interested in restoration measures with other groups who can provide financial or technical resources. The basin coordinator could also play an important role in encouraging other SETPs identified in the workshop.

#### Nonlinear Environmental Thresholds

The final SETP stemmed from participants’ observations that many relationships between environmental variables exhibit threshold behavior. Workshop participants discussed especially the impact of climate change (including warming air and water temperatures and changing precipitation dynamics) as an exogenous force that could drive non-linear environmental responses in the river basin. For example, increases in summer temperatures during low flows and increased brownification are especially stressful to salmonid fishes, whose survival above critical temperature thresholds greatly declines (Sutela et al. 2022). This would be expected to significantly impact biodiversity and recreational usage of the river system. Peatland ecosystems also demonstrate threshold responses to water table levels, with changes beyond critical levels leading to significant shifts in vegetation and biogeochemical processes important for water quality and carbon sequestration (Albert-Saiz et al. 2025). At the same time, it is important to recognize the limitations of the threshold concept. For example, biodiversity loss tends to be gradual, delayed, and multidimensional, rather than neatly corresponding to a single environmental threshold (Hillebrand et al. 2023).

While these ecological tipping points can be challenging to identify and pinpoint, they are crucial to understand as exceeding these thresholds can have direct but non-linear impact on the river basin SES more broadly. Changes in basin hydrology, namely drought and floods, and river temperatures, would also be expected to directly impact other identified tipping points, including (i) land management and restoration, (ii) nature-based tourism, and (iii) demographic and livelihood changes. For example, changes in hydrological regimes and shoulder seasons temperatures have an impact on river water quality and might reduce the effectiveness of water protection and restoration efforts done to improve river health and status. This could lead to a perception that these measures are ineffective, even if they improve water quality conditions relative to a situation where they were not implemented. Special attention should be paid to identify main process drivers that could lead to nonlinear ecological transitions and taking rapid actions to avoid crossing critical thresholds.

## Discussion

### Leveraging SETPs for Environmental Research and Management

By using a conceptual model of SETPs as an analytical frame, we have identified which elements, linkages, and processes are most relevant for the SETP of interest. In general, the SETPs identified were not necessarily unexpected, but rather provided a means to formalize and build out details around general perceptions regarding SES interactions in the basin. In future research, we expect that this would allow us to focus data collection, analysis, and model development on better understanding of those system elements. For instance, coordinated research on SETPs stemming changing perceptions of restoration projects could combine biophysical data collection to quantify their environmental impacts, computational modeling to project their benefits under different future scenarios, and social science research into how different communication and collaboration approaches influence landowner perceptions. This could provide more details into the individual tipping points identified, such as their timing, factors that would impact their likelihood, or relational pathways that clarify causal mechanisms between elements, state variables, and linkages.

This framing can also suggest ways that research activities can align with broader goals of fostering community engagement and participation in restoration efforts. For example, immersive citizen science programs can impact participant awareness, engagement, and action on environmental and conservation issues (Green et al. 2023; Gumiero et al. 2025; McKinley et al. 2024). These factors suggest the power for citizen science to serve as a vehicle for connecting research activities with broader environmental behaviors to create desirable changes. However, it also requires awareness of how different research activities are perceived by the broader public. For example, stakeholders in the Kiiminkijoki have expressed more confidence in empirical research demonstrating the benefits of different management measures rather than computational modeling studies (Räsänen et al. 2024). However, many management solutions require many years before they lead to the desired improvements in outcomes such as water quality or biodiversity and would thus require multidecadal monitoring.

We consider the conceptual model valuable in identifying approaches to integrated environmental policies that aim to address both the physical and social aspects of land use change. For example, in structuring policies to encourage sustainable nature-based tourism in the region, policy makers could not only encourage development in the physical locations that would minimize environmental harm but also design sustainable funding streams for restoration and encourage public education around environmental issues. More generally, policies could be aimed at identifying and taking advantage of “leverage points” where targeted actions could encourage systematic change (Lenton et al. 2022). This presents an alternative to efficiency-focused approaches that aim to maximize the immediate impact of available resources for policy implementation. For example, an efficiency-focused approach might suggest that land use programs should be designed to maximize the areal extent of restoration projects that could be implemented for the funding available. An approach focused on leverage and SETPs would instead try to create seeding conditions for systemic change that would encourage independent landowner adoption of restoration measures. This could include incentivization of projects that provide immediate benefits that can influence public perceptions, as well as broader education campaigns that include peer learning and technical assistance. That said, successful implementation of any program aimed at encouraging positive SETPs would rely on the participation and ownership from the key actors who will be impacted by that program, such as private landowners, to encourage social learning and effective co-design (Ison et al. 2013). However, this likely would require refinement or clarification of conceptual framework so that it is widely accessible and suitable for supporting partnership-level engagement with the relevant stakeholders (Connalin et al. 2017).

### Participant Feedback and Assessment

In addition to participating in the semi-structured elicitation of the tipping points described above, participants were also invited to share feedback on the workshop using a post-event survey and by noting down recommendations as they were working through the activities. However, the response rate for both efforts was low, with seven participants filling out the survey and two providing written feedback. The low survey response rate may be because it was presented at the end of a day-long session and we ran out of time to include it within the day’s activities, or because participants felt they had already had a chance to communicate their perspectives. Because of this low response rate and the unstructured nature of feedback collection, the outcomes presented here should not be viewed as a formal process evaluation. Nevertheless, we include them to provide a sense of how participants viewed the workshop and suggest avenues for more formal evaluation in future research.

The majority of participants who filled out the survey indicated that they found the workshop helpful in gaining a broader and deeper understanding of potential tipping points in the Kiiminkijoki basin (Table S3). The two participants who provided written feedback identified several potential avenues for improving the framework implementation process. Their primary concern was that even with the structure provided by the framework, it was still possible to become overwhelmed by the complexity of the system, and the participants identified several suggestions on how to avoid this. For example, it may be more effective to start with a focus on processes, variables and linkages first, rather than a list of elements that can easily become quite large. Additionally, narrowing the discussion to a more focused lens (e.g., fisheries management, livelihoods, etc.) could make it more feasible to identify relevant processes and linkages without becoming overwhelmed. This outcome was mirrored in observations of small group discussions during the event by the lead author; the groups that focused their tipping points discussion on a sub-issue within river management seemed to more effectively identify and describe those tipping points.

This preliminary feedback suggests the importance of more formal assessment of the conceptual framework and workshop implementation, which would align with calls for better methods for evaluation of transdisciplinary research approaches more generally (Holzer et al. 2018). At a minimum, this could include a more rigorous assessment of participant perceptions on the framework and its individual elements. For example, many participants highlighted the mapping of system linkages as the most challenging part of the system inventory process, as the number and nature of linkages can easily become numerous and highly complex. A focused evaluation of this stage of the process (perhaps by comparing multiple approaches to the elicitation of linkages) could be useful in developing a process that can capture complexity without becoming overwhelmed by it. Ideally, framework evaluation would also include a longer-term investigation into whether workshop outputs have made a meaningful contribution to environmental research and management in the basin.

### Limitations and Areas for Future Research

While the conceptual framework can be used to support collective identification and understanding of prospective SETPs, there are potential areas for improvement and refinement. One key outcome from the workshop is that most prospective SETPs occurred across mechanisms, suggesting that SETP conceptualizations that focus exclusively on feedback processes may miss salient examples of pathways towards systemic change. Additionally, many workshop participants highlighted the mapping of system linkages as the most challenging part of the system inventory process, as the number and nature of linkages can easily become highly complex and overwhelming. More structured approaches to this task that build upon existing methods for social network analysis originating in both the social sciences and physical/mathematical fields (e.g., Lao et al. 2020; Lienert et al. 2013) could thus be beneficial.

One interesting outcome of our workshop was that SETPs identified in the workshop included positive outcomes that could results from targeted interventions (restoration perceptions, nature-based tourism, and the basin coordinator position), undesirable consequences of current environmental trajectories (nonlinear environmental thresholds), as well as dynamics that could unfold in different directions (livelihood change). This speaks to the importance of presenting tipping points as changes that can be both desirable and undesirable. Interestingly, tipping points linked to environmental changes and exogenous forces were the subject of less discussion, despite the rapid climate change in the region (Rantanen et al. 2022) and the fact that exogenous sociopolitical forces have a large impact on how land and water resources are used in European rural areas (Cliquet et al. 2024; Rodríguez-Pose and Bartalucci 2024). Thus, it is important to recognize that the SETPs that emerge from this process are inherently subjective and depend on participant interests, concerns, and level of perceived agency in influencing the processes of interest. Structured methods for system assessment, such as the use of indicator processes aimed at encouraging consideration of broader, system-scale perspectives, could be a valuable way of addressing potential limitations in participant perspectives (Bouckaert et al. 2018). This will also depend on the individuals responsible for collating and interpreting results, pointing to the importance of considering the ultimate goals of the analysis when identifying participants.

## Conclusions

The objective of this research was to operationalize the concept of socio-environmental SETPs using the Kiiminkijoki River Basin as an example application. While this work focused on the Kiiminkijoki specifically, the basin is representative of many areas in the Nordic countries where land management decisions must balance multiple environmental and economic goals, while also being cognizant of individual landowner rights. We demonstrated how a previously developed SETP conceptual framework can be used to provide a consistent vocabulary to develop plausible pathways by which tipping could occur. The identified SETPs in our Kiiminkijoki case include feedback loops related to perceptions of land restoration and management, nature-based tourism development, demographic and livelihood changes, the establishment of a new basin coordinator position, and nonlinear environmental relationships. While more in-depth investigation of these pathways is beyond the scope of this research, we argue that the framework outlined here could provide a structured means for organizing interdisciplinary research into different mechanisms of rapid change in SES. By highlighting where connections between social and environmental elements have the potential to be most impactful, the approach can also help identify areas where integrated management of social and environmental processes could be used to potentially create systemic-level change. Using the framework demonstrated in this work can help to structure empirical research on socio-environmental tipping points so that the current enthusiasm for the concept can be leveraged to obtain actionable insights into complex pathways of change in SES.

## Supplementary information

Supplementary Material

## Data Availability

No datasets were generated or analyzed during the current study.
