# Supplementary material for: Operationalizing the Concept of Socio-environmental Tipping Points in River Basin Management
Source: Environ Manage. 2026 Aug 21;76(9):284. doi: 10.1007/s00267-026-02585-z (PMC13498642; doi:10.1007/s00267-026-02585-z)
Supplement: Supplementary file 1 [file 267_2026_2585_MOESM1_ESM.pdf]

S.1 Conceptual Framework Overview and Rationale

Two important principles of SESs are that 1) they are defined more by the interactions between their individual components rather than the components themselves; and 2) their behavior is generated through dynamic processes (Preiser et al., 2018). Clearly accounting for these relationships and processes is crucially important in characterizing SESs in a coherent way. Thus, the conceptual framework is organized in a fashion that can clearly differentiate between the tangible *elements* that exist within the system, the *state variables* that describe those elements (these state variables could also be considered attributes), and the *linkages* that describe how different elements interact and influence each other. These first three components of the framework offer a means of describing the system at a single point in time. To incorporate descriptions of change, the dynamics that influence system behavior through time are organized by distinguishing *internal processes* which can be meaningfully influenced by systems actors and *exogenous forces* which cannot. The division between processes and exogenous forces is not objective and clear-cut, as many processes may be partially influenced by actors in the system and a defining feature of socio-environmental system is the fact that they are "radically open" and rarely have a clear set of objective boundaries (Preiser et al., 2018). However, there is a value in distinguishing changes that emerge from population processes and those that emerge from changes in system inputs, as these have different implications for feasible management strategies (Currie et al., 2023).

After the SES inventory has been developed, it can be reviewed for three potential tipping mechanisms. Feedback processes occur when the drivers of a process (state variables that contribute to the process) are impacted by the same change. Feedback processes can be stabilizing (i.e., negative feedback loops) or amplifying (i.e., positive feedback loops). Stabilizing feedback processes will typically keep a system in a relatively steady condition, whereas amplifying feedback processes can have the opposite effect. It should be noted that "positive" within the context of feedback processes refers to whether or not the process is amplifying or self-reinforcing. In this sense, positive feedback loops could be desirable or undesirable. For clarity, we thus refer to desirable tipping points as being intentional or desirable, rather than "positive."

Cascading linkages occur when the structure of linkages across SES elements create conditions where marginal changes in the state variable of one element impacts multiple additional elements. For example, changes to a highly central institution or business have a greater potential to cascade into system-wide impacts than changes to a peripheral element. Linkages between elements can also create "domino effects" such as the trophic cascades (Ripple & Beschta, 2012). The linkages assessment in this conceptual framework aims to provide an initial means of characterizing system connections that could lead to rapid or cascading changes. This initial assessment could be strengthened considerably through the integration of formal social network analysis methods (e.g., Lienert et al., 2013). Nonlinear relationships between state variables can lead to threshold points where a slight change in one state variable causes a large change in another. For example, exceeding certain environmental conditions can cause species collapse, relationships between elevation and land area lead to nonlinear flood risk distributions, and business profitability often grows nonlinearly with scale.

These different mechanisms are not necessarily exclusive of each other, and in many examples could occur simultaneously. More discussion on tipping mechanisms can be found in Shortridge et al. (2024).

S.2 Complete System Inventory Components

**Elements**	**Descriptive state variables**
Private Landowners	• Population: size, demographics including age structure<br>• Residence (local or remote)<br>• Area of land operated<br>• Income: amount, sole or partial income from land<br>• Financial and technical resources<br>• Utilization of services<br>• Level of participation in community efforts<br>• Values (relative value of environment, economy, etc.)<br>• Perceptions (e.g., perceived feasibility of new technologies)
Full time residents	• Population: size, demographics including age structure<br>• Utilization of services<br>• Level of participation in community efforts<br>• Values (relative value of environment, economy, etc.)<br>• Perceptions (e.g., current state of river)
Seasonal residents (e.g., summer cottage owners)	• Population: size, demographics<br>• Time spent in watershed<br>• Utilization of services<br>• Level of participation in community efforts<br>• Values (relative value of environment, economy, etc.)<br>• Perceptions (e.g., current state of river)
Visitors/tourists	• Number of visitors<br>• Values (e.g., importance of nature)<br>• Types of visit (e.g. fishing, winter sports, etc.)
Community groups, civil society organizations	• Number of members<br>• Goals<br>• Resources available (money, expertise, facilities)
Churches and other religious organizations	• Number of members<br>• Level of participation in community efforts
Municipal governments (Oulu, Pudasjärvi, Puolanka, Utajärvi)	• Mandate and responsibilities<br>• Goals<br>• Resources (financial, expertise, etc.)

Regional administrations (e.g., ELY Centre; Council of Oulu Region; Regional Council of Kainuu)	• Mandate and responsibilities<br>• Goals<br>• Resources<br>• Participation in regional and national initiatives (e.g., Priodiversity LIFE)
Institutional Landowners (especially Metsähallitus but also parishes, municipalities)	• Area of land operated<br>• Management practices<br>• Income and role of forestry in income<br>• Resources (financial, staff, etc.)<br>• Mandate, responsibilities, and goals<br>• Policy constraints<br>• Level of participation in community efforts<br>• Services provided
Educational and advisory organizations (ProAgria, MTK, forestry associations, Forest Centre, Tapio)	• Resources (financial, staff, etc.)<br>• Goals<br>• Number of participants
Research organizations (University of Oulu, Natural Resources Institute Finland, Finnish Environment Institute)	• Resources (financial, staff, facilities, etc.)<br>• Goals<br>• Current projects
Public schools	• Number of spaces for students<br>• Perceived quality of education
Farms	• Income, including subsidies or participation in carbon markets<br>• Activities (e.g. livestock, crops, reindeer herding)<br>• Management practices<br>• Financial and technical resources<br>• Employees and labor available
Timber companies	• Income (including subsidies or participation in carbon and biodiversity markets)<br>• Management practices<br>• Financial and technical resources<br>• Employees and labor available
Natural resources businesses (e.g., buyers, processors, paper and pulp industries)	• Income<br>• Financial and technical resources<br>• Employees and labor available

Land and water extraction companies, (e.g., water supply, sand and gravel extraction, mining)	• Areal extent of operation<br>• Practices and management
Energy companies, including electricity production (bioenergy, wind, solar, etc.)	• Number and type of companies (fuel type)<br>• Location<br>• Income<br>• Employees and labor available
Reindeer herding	• Land operated<br>• Income (including subsidies)<br>• Participation in community efforts
Other businesses	• Income<br>• Financial and technical resources<br>• Employees and labor available
Interest and advocacy organizations (e.g., The Central Union of Agricultural Producers and Forest Owners, Forestry Associations)	• Goals<br>• Financial and technical resources<br>• Participation in community efforts
River, streams, lakes	• Pollutant and nutrient concentrations (e.g., iron, humic compounds, acidity)<br>• Flow rate regime, including seasonal variations<br>• Conservation or protection status (e.g., Natura 2000)<br>• Ecological status (EU Water Framework Directive)
Peatlands	• Areal extent<br>• Extraction activities (current and historic)<br>• Conservation or protection status<br>• GHG emissions
Mineral soil areas	• Hydrology patterns<br>• Long-term fertility
Farms	• Areal extent<br>• Crops grown<br>• Livestock supported<br>• Management practices in place<br>• Rate of nutrient loading<br>• Drainage system<br>• Conservation or protection status (e.g., biodiversity hotspot, EU Natura 2000, national or private nature protection areas)
Forests	• Areal extent<br>• Tree types<br>• Harvesting levels and practices<br>• Protection/conservation status<br>• Carbon balance

	• Rate of nutrient loading<br>• Drainage system
Restored areas	• Areal extent<br>• Restoration conditions and characteristics<br>• Rate of nutrient loading or retention
Transportation infrastructure	• Mileage and coverage of area<br>• Condition, reliability (especially regarding flooding.)
Species (salmon, trout, pearl mussels, etc., but also pathogenic and invasive species, cyprinids, etc.)	• Population<br>• Health (disease levels, reproduction rates, etc.)<br>• Native versus invasive

	Driving elements		Impacted elements	
**Process Name**	**Elements**	**State Variables**	**Elements**	**State Variables**
Construction of restoration and protection measures	Farmed and forested land	Productivity levels, soil conditions (mineral or peatland)	Restored and protected land	nutrient retention, carbon and GHG balance, biodiversity
	Forest and farm landowners (private)	current income from farming and forestry, financial resources, technical understanding of options, perceptions of restoration benefits and feasibility	Forests and farm landowners (private)	Perceptions of watershed health, perceptions of benefits and feasibility of restoration and protection projects
	Forest and farm landowners (institutional)	Income and role of forestry in income, resources, policy constraints, objectives	Residents	Perceptions of watershed health
	Former peatland extraction site landowners	financial resources, technical understanding of options, perceptions of feasibility		
	Municipalities and regional administration	Perceptions of feasibility, constraining or encouraging regulations, presence and resources available for incentive programs		
	Researchers	Resources, goals, funding, projects		
	Educational and advisory organizations	Resources, goals, number of participants		
	Related industries (e.g., construction, materials)	Income, financial and technical resources, labor available		

Farming and forestry management changes, including formal land conservation	Farmed and forested land	Productivity levels, soil conditions (mineral or peatland)	Farm and forested land	Nutrient retention, carbon and GHG balance, biodiversity
	Farm and forestry landowners (private)	Current income from farming and forestry, financial resources, technical understanding of options, perceptions of benefits and feasibility, values	Farm and forestry landowners (private)	Income from farming and forestry, technical understanding of options, perceptions of benefits and feasibility
	Farm and forestry landowners (institutional)	Income and role of forestry in income, resources, policy constraints, objectives	Farm and forestry landowners (institutional)	Perceptions of benefits and feasibility
	Municipalities and regional administration	Constraining or encouraging regulations, presence and resources available for incentive programs	Agricultural and forestry related businesses and industries	Income, perceptions of value
	Researchers	Resources, goals, funding, projects		
	Educational and advisory organizations	Resources, goals, number of participants		
	Agricultural and forestry related businesses and industries	Income, financial and technical resources, labor available		
Economic development	Land	Productivity levels, prices	Land	Nutrient/pollution runoff, carbon/GHG balance
	Landowners (private)	Current land-based income, values (connection to locality), perception of future income	Businesses	number, type of services, income, jobs provided

	Landowners (institutional)	Income and role of land in income, objectives, policy constraints	Residents	Income levels, employment
	Residents	Income levels, values, employment	Municipalities	Resources (including tax base)
	Municipalities	Constraining or encouraging regulations, presence and resources available for incentive programs		
	Businesses	Number, type of services, income, financial and technical resources, labor available		
Infrastructure investment (energy, transportation, etc.)	Infrastructure facilities	Current levels of service, demand for services	Infrastructure facilities	level of service, income
	Landowners (private)	Current land-based income, values (connection to locality), perception of future income	Land	Prices, existing use patterns
	Landowners (institutional)	Income and role of land in income, objectives, policy constraints	Residents	Perceptions of values and benefits, jobs and employment
	Land	Productivity levels, prices, existing use patterns	Municipalities	Resources (including tax base)
	Residents	Demand for services, perceptions of value and benefits, values		
	Municipalities	Constraining or encouraging regulations, presence and resources available for incentive programs		
	Supporting industries (e.g., construction)	Income, financial and technical resources, labor available		

Local demographic change	Land	Productivity levels, prices, existing use patterns	Land	Prices, existing use patterns, nutrient/pollution runoff, carbon/GHG balance
	Landowners (private)	Current land-based income, values (connection to locality), perception of future income	Landowners (private)	Values (connection to locality)
	Landowners (institutional)	Income and role of land in income, objectives, policy constraints	Landowners (institutional)	Demand for services
	Residents	Demand for services, perceptions of community, employment, values	Residents	Local values, norms and priorities (including relationship to nature)
	Municipalities	Service provision and availability, constraining or encouraging policies	Municipalities	Resources (including tax base)
	Businesses	Number, type of services, income, financial and technical resources, labor available	Businesses	Labor available, types of services
Regulatory change	Landowners (private)	Land-based income, values, perceptions	Land	Land use patterns and management, productivity
	Landowners (institutional)	Income and role of land in income	Landowners	Income, perceptions and values
	Residents	Employment and income, values, perceptions	Residents	Perceptions and values (e.g., collective versus individual values)
	Municipalities, regional administration	Objectives, mandates/responsibilities	Businesses	Income
	Businesses (e.g., forest and other natural resources industries)	Income, perceptions, values	Municipalities	Resources (including tax base)

S.3 Summary of Workshop Participants and Feedback Results

The transdisciplinary researcher-practitioner workshop was attended by 17 researchers and practitioners from Finnish universities, government research institutes, and regional and municipal administrative offices. The participants had a background in a range of disciplines, including hydrology, ecology, anthropology, geography, engineering, and environmental and water resources management. Participants were drawn from multiple university, governmental, and non-governmental organizations as follows:

- University of Oulu Research Units
    - Geography
    - Water, Energy, and Environmental Engineering
    - History, Culture, and Communication Studies
    - Ecology and Genetics
- Natural Resources Institute Finland (LUKE)
- Geological Survey of Finland (GTK)
- Finnish Environment Institute (SYKE)
- Regional Economic Development, Transport, and Environment (ELY) Centre
- Micropolis Green Development and Marketing Company

A table summarizing the topical expertise and professional role of each participant is included in Table S3:

Table S3: Workshop participant areas of expertise. Role indicates whether the individual is more involved in research (R) or practice (P).

ID	Area of Expertise	Role
1	Spatial methods, resilience, environmental systems	R
2	Water quality, hydrological processes, catchment management	R
3	Human-environment interactions, rural communities	R
4	Rural Anthropology	R
5	Migratory fish, regulated rivers	R
6	Forest genetics, tree breeding	R
7	Peatland restoration, remote sensing	R
8	Nature-based solutions and water quality	R
9	Sustainable tourism and environmental change	R
10	Stakeholder-informed biodiversity monitoring	R
11	Environmental anthropology	R
12	Anthropology, environmental social science	R
13	Ecology, genetics, citizen science	R
14	River basin coordination and management	P
15	Water Resources Management	P
16	Green and sustainable economic growth	P
17	Freshwater ecology and conservation	P

In addition to tipping point identification, participants were also invited to share feedback on the workshop using a post-event survey and by noting down comments during the workshop. However, the response rate for both efforts was low, with seven participants filling out the survey and two providing written feedback. The low survey response rate may be due to the fact that it was presented at the end of a day-long session and we ran out of time to include it within the days activities. Because of this low response rate and the unstructured nature of feedback collection, the outcomes presented here should not be viewed as a formal process evaluation. Nevertheless, we include them to provide a sense of how participants viewed the workshop and suggest avenues for more formal evaluation in future research.

Table S4 presents the results of a Likert-scale questionnaire where participants ranked their agreement with five statements on a scale of 1-5, where 5 indicates strong agreement and 1 indicates strong disagreement. In general, survey responses indicated that the participants found the workshop helpful in gaining a broader and deeper understanding of potential tipping points in the Kiiminkijoki basin. Responses related to identifying transdisciplinary connections (Q2) and tipping points (Q4) were slightly higher than responses related to generating new

perspectives (Q1), identifying avenues for individual contributions (Q3), and making sense of complex tipping points (Q5).

Table S4: Post-workshop survey results. The answers were given on a Likert scale with 5 indicating strong agreement and 1 indicating strong disagreement.

**Question**	**Average Score**
The workshop helped me look at issues in the Kiiminkijoki in a new way	3.75
The workshop helped me identify connections between my field and others	4
The workshop helped me identify ways that I could contribute to larger, interdisciplinary questions	3.88
The workshop helped me identify potential tipping points in the Kiiminkijoki	4.13
The workshop helped me make sense of potential tipping points in the Kiiminkijoki	3.75

The comments received through the unstructured feedback process were as follows:

- Some people in the room (myself included) had little knowledge of the Kiiminkijoki basin specifics prior to the meeting. An intro background document or presentation at beginning would have helped.
- I don't the think the process had enough constraints to prevent overwhelm by complexity. Starting with too many elements and no specific lens to start with left too many links to consider.
- To get each group to focus on tipping points, the sessions needs to be engineered to make variables and transitions visible earlier. Example: make groups pick a lens to work through (fish, livelihoods)
- It would have helped to make groups start by making a diagram of elements and linkages, but with a hard limit on number of each. That makes maps possible, and ID of tipping points within that.
- Another possible alternative would be to make the working sessions focus on making different maps of elements and linkages, with identified tipping points in each.
- I think that the connections between different elements of the SES are more important to recognize than the elements themselves. Focusing more on identifying processes and links might be more useful

- Identifying which type the tipping points are was difficult. Maybe they should be thought more as multidimensional things rather than singular mechanisms. Emphasizing the connections would be better.
- Additional prompts would be useful to encourage thinking about non-local actors whose activities impact the basin (eg paper factories which will harvest from the basin; climate change; invasive species which are “arriving” etc)
- It is important to recognise that one category such as “landowners” actually hide a great variety of motivations & behaviours, eg metsähallitus can “act as one” while absentee landowners will have different patterns of behaviour
- An overall observation / prompt: what would it be like if instead of actors we started with processes / relations? What would it mean to map the basin as a system of relations (growing, drainage, human livelihoods etc) & then map the actors who impact processes? Just a thought. (Eg what do local humans “need to live”, what do forests “need to live” etc...)
- With regards to positive tipping points – it strikes me how much we are caught in the status quo here; it is easy to identify largely destructive/reactive actors & think how we could mitigate the evil they do – but so much harder to think what social infrastructure may exist to tip the system into a new state. Eg degrowth, ecological living or other low-resource livelihoods oriented groups. These are not on our radar at all...

S.4 Supplementary References

Currie, T. E., Borgerhoff Mulder, M., Fogarty, L., Schlüter, M., Folke, C., Haider, L. J., Caniglia, G., Tavoni, A., Jansen, R. E. V., Jørgensen, P. S., & Waring, T. M. (2023). Integrating evolutionary theory and social–ecological systems research to address the sustainability challenges of the Anthropocene. *Philosophical Transactions of the Royal Society B: Biological Sciences*, *379*(1893), 20220262. https://doi.org/10.1098/rstb.2022.0262

Lienert, J., Schnetzer, F., & Ingold, K. (2013) Stakeholder analysis combined with social network analysis provides fine-grained insights into water infrastructure planning processes. Journal of Environmental Management, 125, 134-148.

Preiser, R., Biggs, R., De Vos, A. & Folke, C. Social-ecological systems as complex adaptive systems: organizing principles for advancing research methods and approaches. Ecology and Society 23, (2018).

Ripple, W. J., & Beschta, R. L. (2012). Trophic cascades in Yellowstone: The first 15 years after wolf reintroduction. Biological Conservation, 145(1), 205–213. https://doi.org/10.1016/j.biocon.2011.11.005

Shortridge, J. E., Bukvic, A., Mitchell, M., Goldstein, J., & Allen, T. (2024). Characterizing Climatic Socio-Environmental Tipping Points in Coastal Communities: A Conceptual Framework for Research and Practice. Earth’s Future, 12(7), e2023EF004123. https://doi.org/10.1029/2023EF004123

S.5 Workshop Slides

The slides presented to provide an overview of the conceptual framework during workshop activities are included below.

Investigating the potential for socio-environmental tipping points related to climate and land use change in Northern Finland

University of Oulu

Science Garden

14 January, 2025

1

Acknowledgements

- Research Support: United States Fulbright Program and Fulbright Finland Foundation
- Workshop Sponsorship: U Oulu Digital Waters Flagship and Frontiers of Arctic and Global Resilience (FRONT) Research Programme
- Kiiminkijoki guidance and advice: Aleksi Räsänen and Hannu Martilla
- Logistical support: Hanna Holappa

FULBRIGHT PROGRAM ★ ESTABLISHED 1946 ★

FULBRIGHT FINLAND FOUNDATION

Digital Waters

FRONT UNIVERSITY OF OULU

2

Morning Agenda

Time	Activity
9:00 – 9:20	Introduction to project and tipping points
9:20 – 10:00	System inventory refinement: elements and state variables
10:00 – 10:15	Coffee break
10:15 – 10:50	System inventory refinement: linkages
10:50 – 11:30	System inventory refinement: processes and exogenous forces

3

Motivation for Conceptual Framework

- Recent research on climate change and socio-environmental tipping points

- Local relevance: rapid sea level rise, "nuisance flooding"

- Interdisciplinary team interested in investigation the potential for tipping points in our home state of Virginia

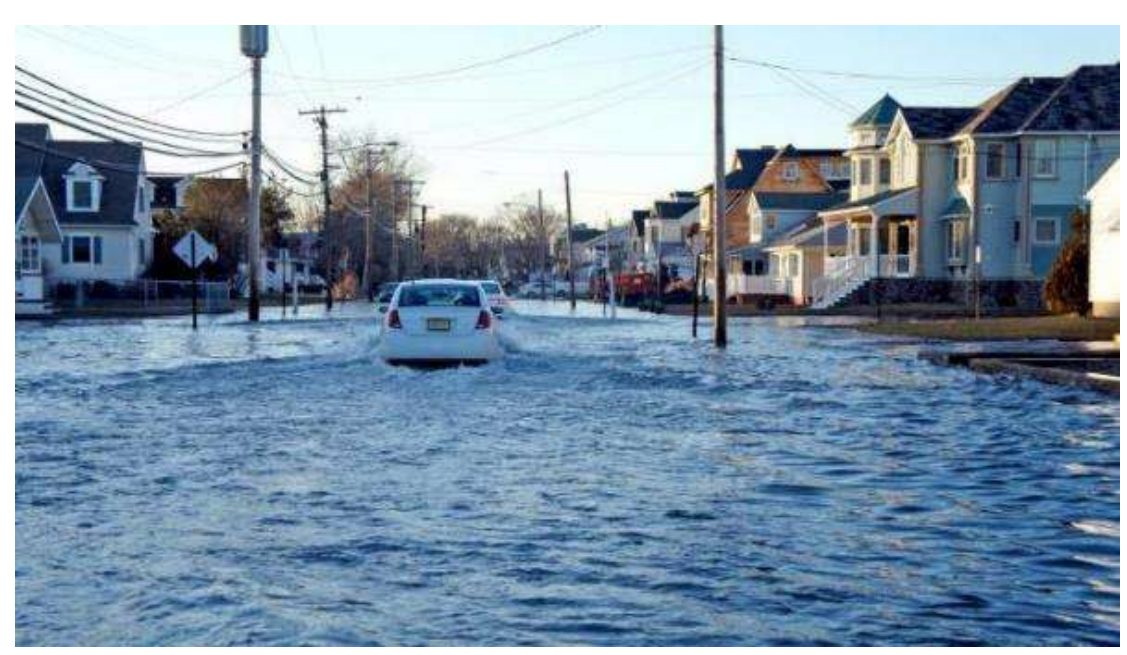

Gradual changes in sea-level rise → Rapid, significant community impacts

4

Socio-Environmental Tipping Points

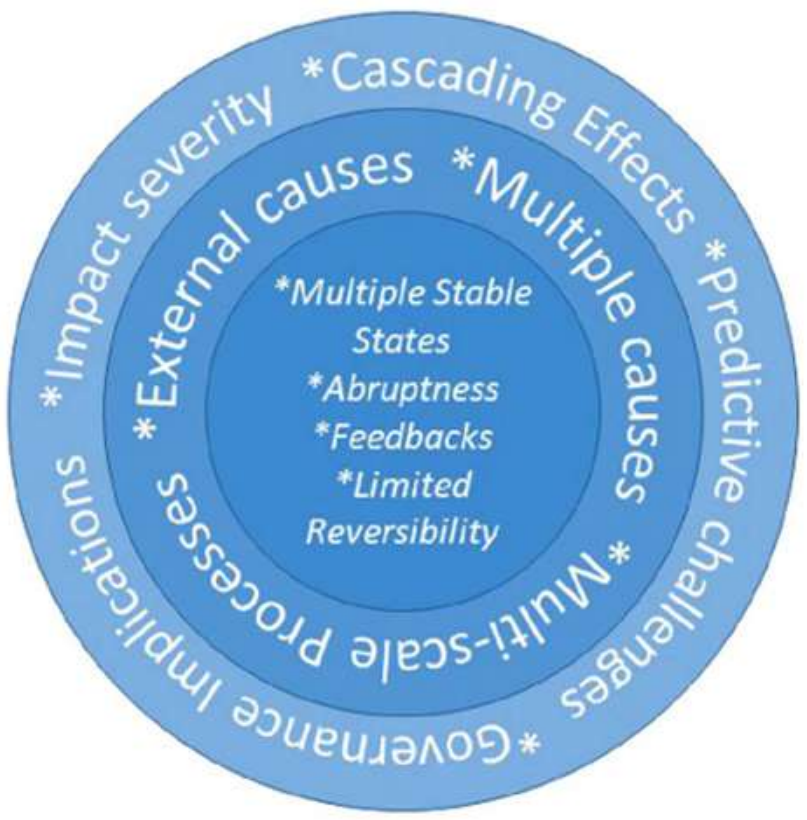

- Conceptual elements: Rapid or abrupt changes to different state
- Examples (van Ginkel et al., 2020):
  - Collapse of winter sports tourism
  - Farmland abandonment
  - Sea-level rise induced migration
- Challenges:
  - Multiple, i…
  - Highly …
  - Numerous …
  - Multiple epist… …ages

"Stranded in complexity"

Figure from Milkoreit et al., 2018. Defining tipping points for social-ecological systems scholarship—an interdisciplinary literature review

5

Descriptive Framework: SES Inventory

Elements:
What exist within the system?

State variables:
Describe element characteristics

Linkages:
How are elements connected with each other?

Internal Processes:
How do elements and state variables change through time?

Exogenous Forces

6

Tipping Mechanisms

- Feedback processes
- Cascading linkages
- Nonlinear relationships between state variables

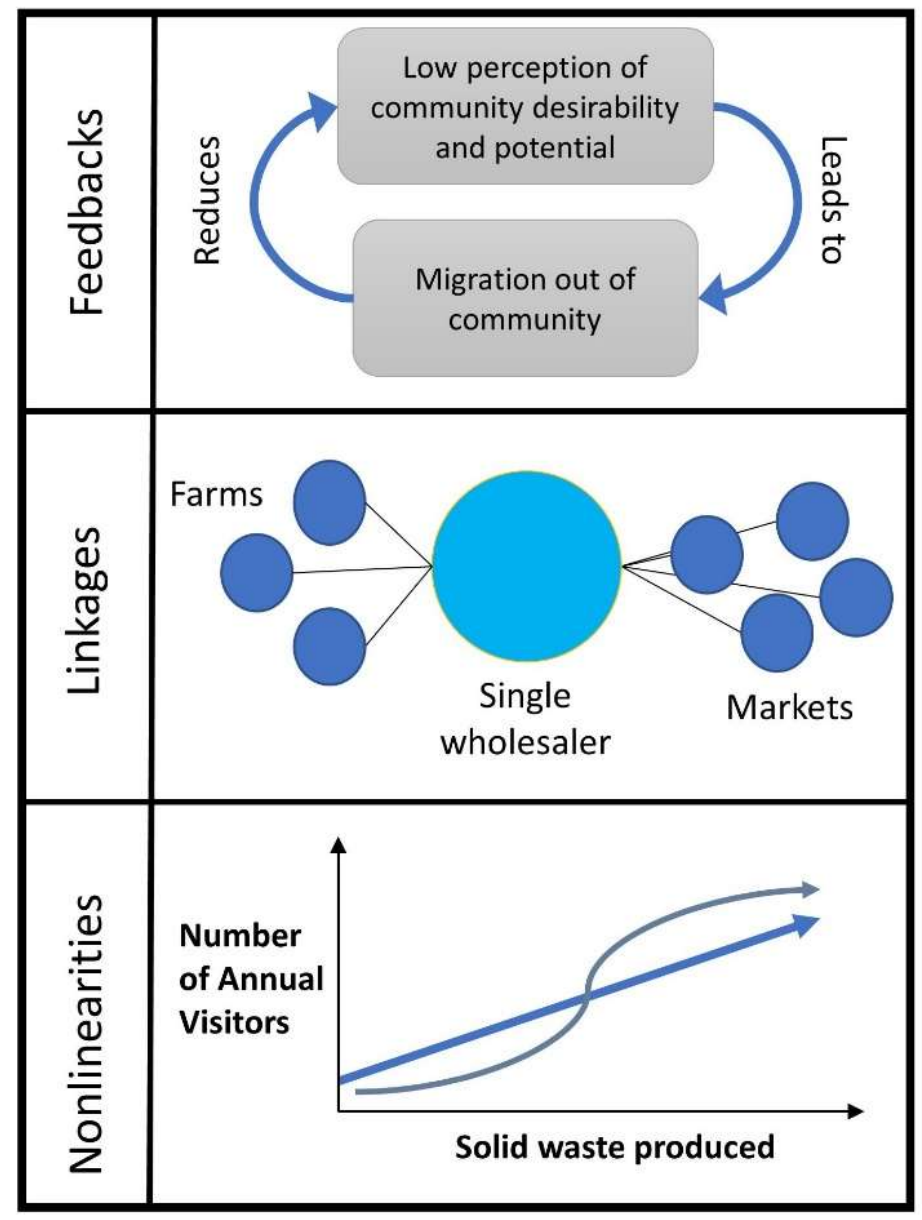

7

Why Kiiminkijoki?

- Northern parts of the globe are warming at four times the global average.

- Finland's ambitious climate policies – potential implications for forestry, natural resources

- Kiiminkijoki: building on outcomes and activities from MATKI project and basin roadmap.

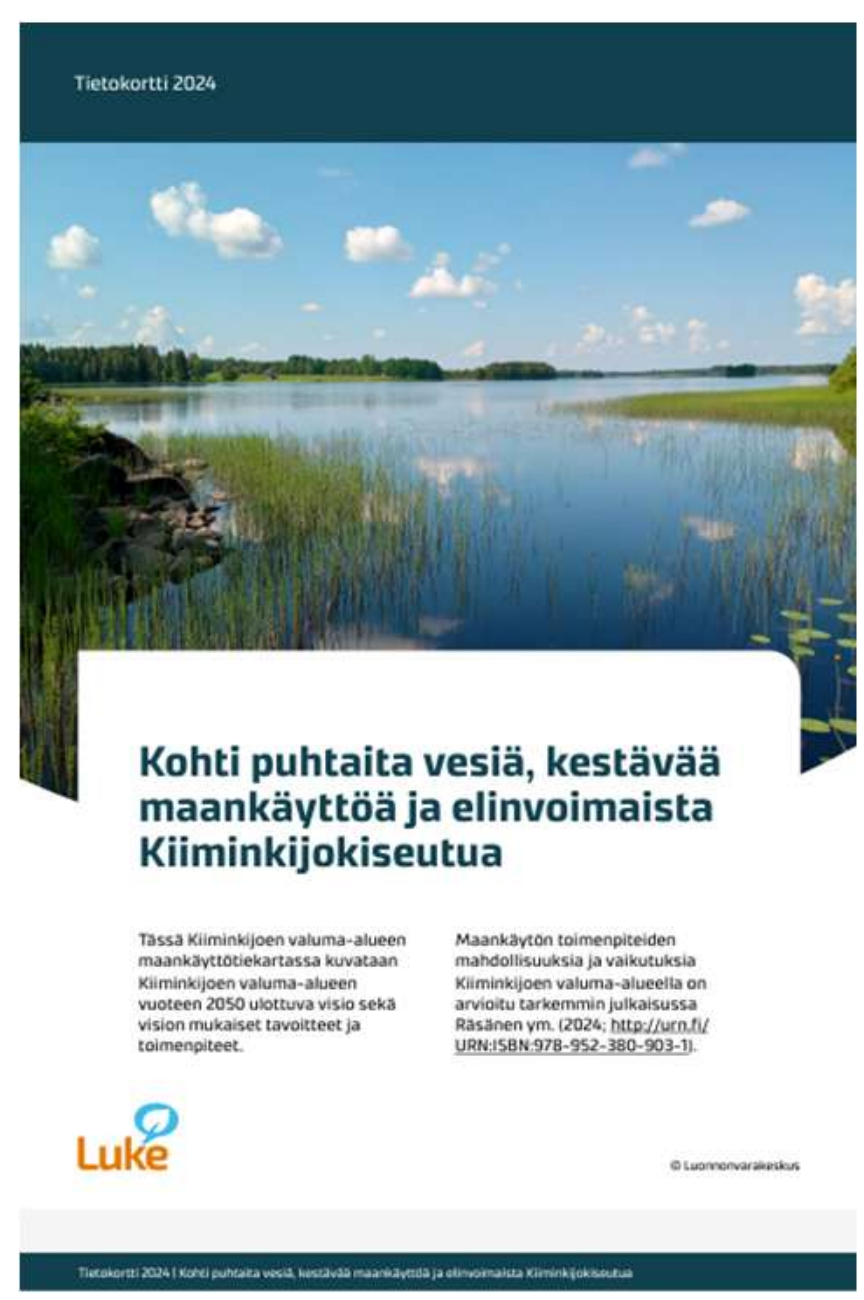

Tietokortti 2024

Kohti puhtaita vesiä, kestävää maankäyttöä ja elinvoimaista Kiiminkijokiseutua

Tässä Kiiminkijoen valuma-alueen maankäyttötiekartassa kuvataan Kiiminkijoen valuma-alueen vuoteen 2050 ulottuva visio sekä vision mukaiset tavoitteet ja toimenpiteet.

Maankäytön toimenpiteiden mahdollisuuksia ja vaikutuksia Kiiminkijoen valuma-alueella on arvioitu tarkemmin julkaisussa Räsänen ym. (2024; http://urn.fi/URN:ISBN:978-952-380-903-1).

Luke

© Luonnonvarakeskus

Tietokortti 2024 | Kohti puhtaita vesiä, kestävää maankäyttöä ja elinvoimaista Kiiminkijokiseutua

8

Goals for the Day

Overall goal: identify potential socio-environmental tipping points related to land-use and climate change in the Kiiminkijoki basin

Objectives:

1) refine and expand a system inventory for the basin

2) review processes and linkages within this system inventory for potential tipping

3) prioritize potential tipping points based on their perceived impact and likelihood.

As a secondary goal, we will identify data needs and research priorities that could improve understanding and management of the prioritized tipping points.

9

Structure of the Day

- Morning: Advancing and Refining SES Inventory for Kiiminkijoki basin
- Afternoon: Reviewing the SES Inventory to identify potential tipping points
- Keep in mind:
  - No one knows everything, all can contribute something!
  - Please record ideas on paper or shared documents
  - Busy day – please be thoughtful of allowing time for others to share
- Looking ahead:
  - Synthesis of notes and outcomes from workshop
  - Interest in further collaboration (manuscripts, proposals, etc.) can be demonstrated through survey at the end of the day
- Questions?

10

System Inventory Refinement

- Goals:
  - Identify and describe the social and environmental agents, processes, and relationships that could impact sustainability and resilience in the basin as they relate to land-use
  - Build collective understanding across the larger group in a structured way
- Not a goal: create an exhaustive list of everything in the system
  - Do be inclusive, but don't try to capture every single thing

11

Working files – two options

- Pen and paper
- Shared online documents

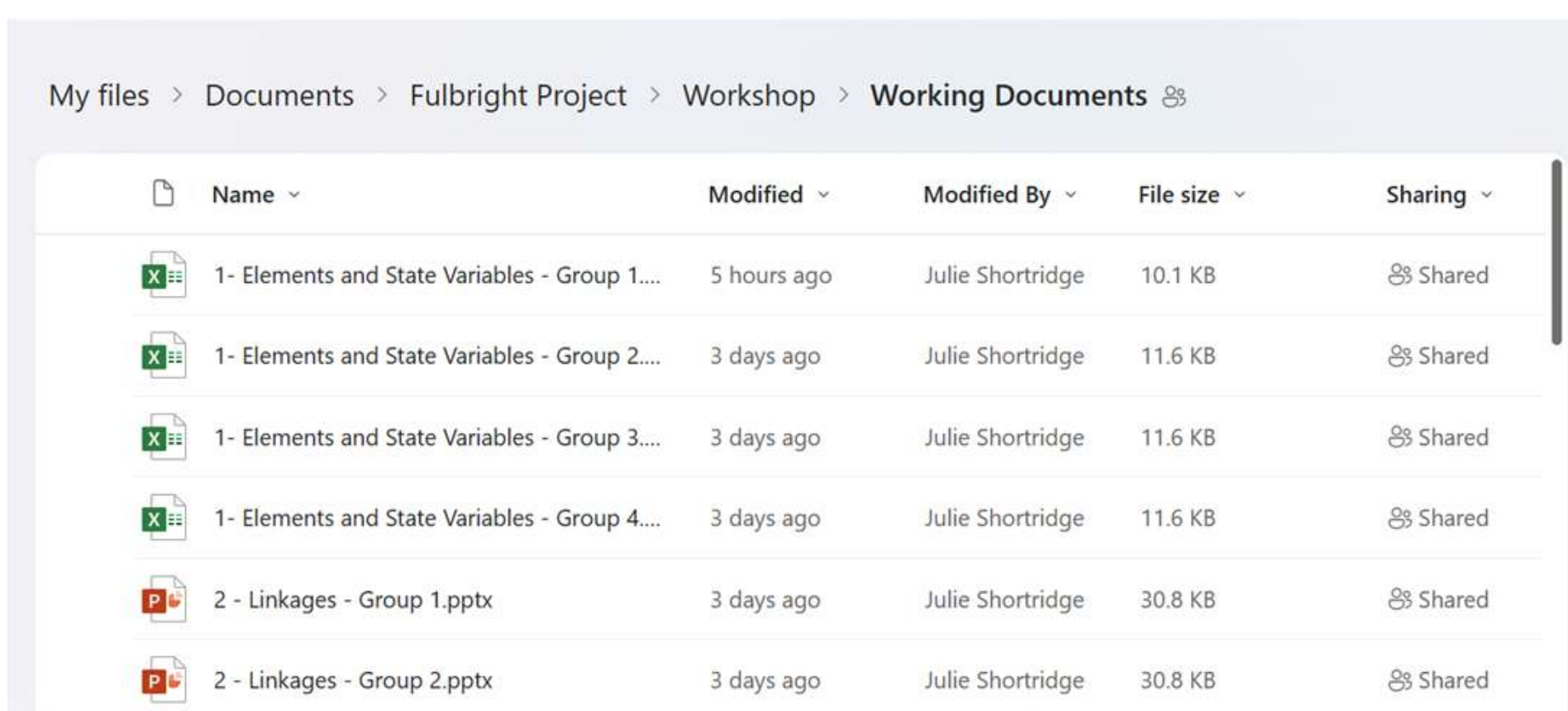

12

System Inventory: Elements and State Variables

Small group discussions: 9:35 – 10:00

- Elements: the actors, institutions, and physical assets that are present in the system of interest and influence its resilience and sustainability
- State Variables:
  - Describe elements in terms of physical, institutional, economic, and socio-cultural characteristics.
  - Can be quantitative or qualitative
  - Could conceivably be measured
- Goals for refining existing list of elements and state variables:
  - Broadening: what is missing from the list?
  - Specifying: for example, “landowners” could be replaced with residents, farmers, foresters, etc.

13

Elements and State Variables

Domain	Example Elements	Example State Variables
Population	• Individuals, households<br>• Visitors or temporary residents	• Population size and demographics<br>• Values and norms, knowledge, perceptions, efficacy<br>• Degree of cohesion/division, equity/inequity
Institutional	• Government agencies<br>• Churches<br>• Schools<br>• Health care facilities<br>• Community organizations	• Capacity and resources<br>• Influence<br>• Number of members<br>• Organizational values and goals
Economic	• Businesses<br>• Banks	• Business community characteristics<br>• Employment profile<br>• Tax base<br>• Availability of goods and services
Physical	• Land cover<br>• Building stock<br>• Shoreline<br>• Built and natural infrastructure<br>• Public spaces	• Distribution of land use<br>• Age and condition of built assets<br>• Shoreline characteristics (e.g., hardened or not)

14

System Inventory Refinement

Small group discussions: 10:35 – 10:55

- Linkages: connections between elements within the SES
  - Can be conceptualized as a change in the state variable of one element impacting the state variable of another.
  - Can encompass many different types of relationships (physical connections, financial interactions, cultural influences, and regulatory authority).
  - Can include one-directional or two-directional influences
- Goals for refinement:
  - Broadening: what is missing? Where do new elements fit in?
  - Specifying: What is the nature of this linkage?
    - Degree and direction of influence, control, or reliance
    - Physical, financial, institutional, or a combination?

15

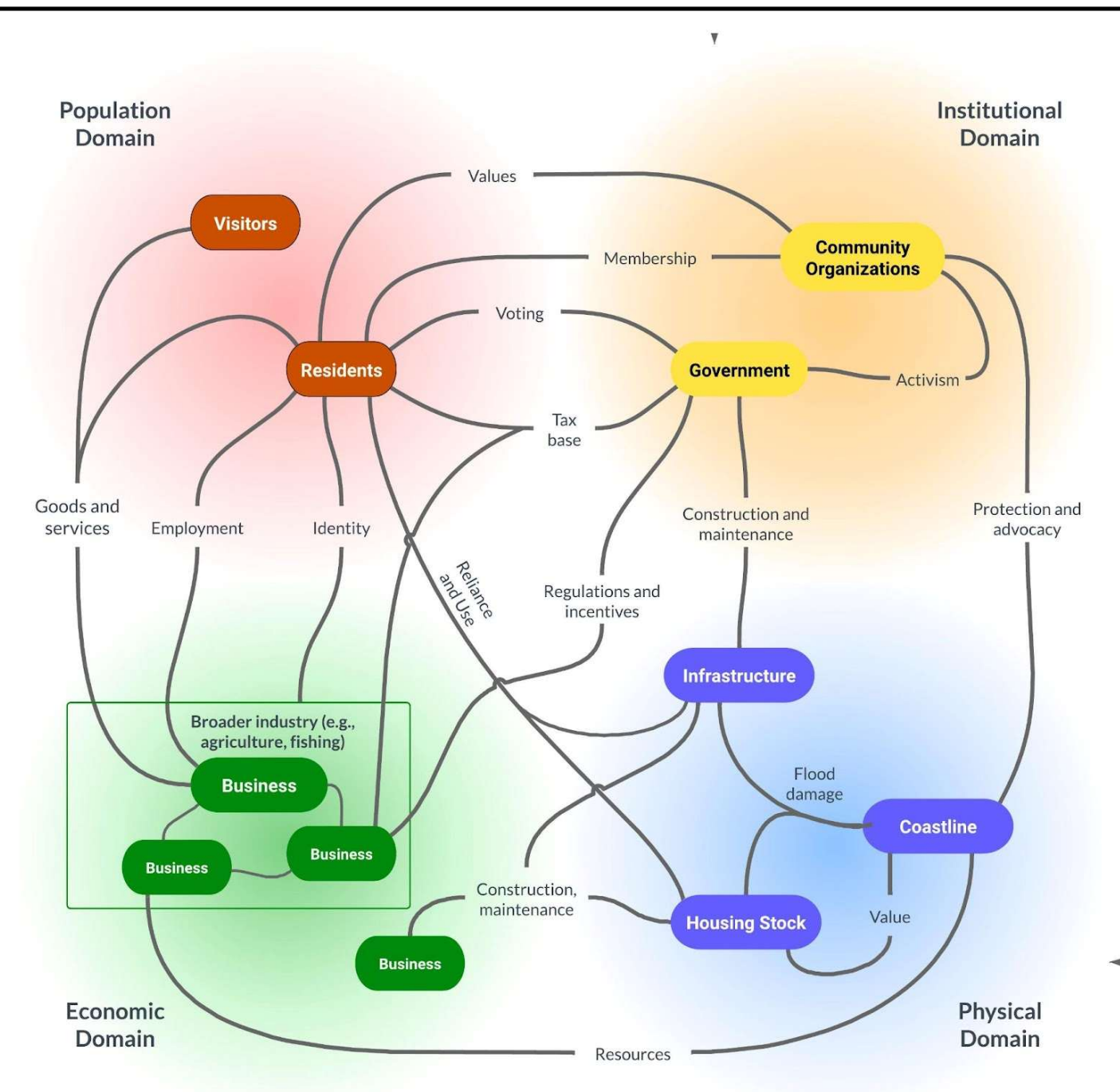

16

Processes and Exogenous Forces

Internal Processes
- Internal mechanisms by which elements and state variables change.
- Examples: in/out migration, land use change, economic growth

Exogenous Forces
- External factors that influence elements and state variables
- Examples: Sea level rise, broad-scale economic drivers, top-town governmental programs

Key difference:
agency and influence of actors in system

17

System Inventory Refinement

Small group discussions: 11:15 – 11:35

- Processes: internal mechanisms by which elements change
  - Changes in state variables
  - Addition and removal of linkages or even the elements themselves
- Exogenous forces: Act on and influence the SES, but are beyond the bounds of control and influence by SES actors
- Goals for refinement:
  - Broadening: what is missing? Where do new elements fit in?
  - Specifying:
    - Processes: What elements are involved? What drivers contribute to a process? What are the outcomes and results of that process?
    - Exogenous Forces: More details on types of forces (e.g., what regulations?)

18

Time to eat!

19

Afternoon Agenda

Time	Activity
12:30 – 12:50	Tipping point mechanisms overview
12:50 – 14:00	Prospective tipping point identification
14:00 – 14:10	Coffee break; tipping point ranking
14:10 – 14:45	Analysis of prospective tipping points
14:45 – 15:00	Closing remarks

20

Tipping Point Mechanisms

How can rapid, significant change happen in socio-environmental systems?

1. Feedback processes
2. Cascading linkages
3. Nonlinear relationships

21

Feedback Processes

- All processes have drivers (elements and state variables that contribute to the process) and outcomes (elements and state variables are impacted by that same change.
- If a driver of a process is impacted by that same process, this is a feedback loop
- Feedback loops can be negative (stabilizing) or positive (amplifying)

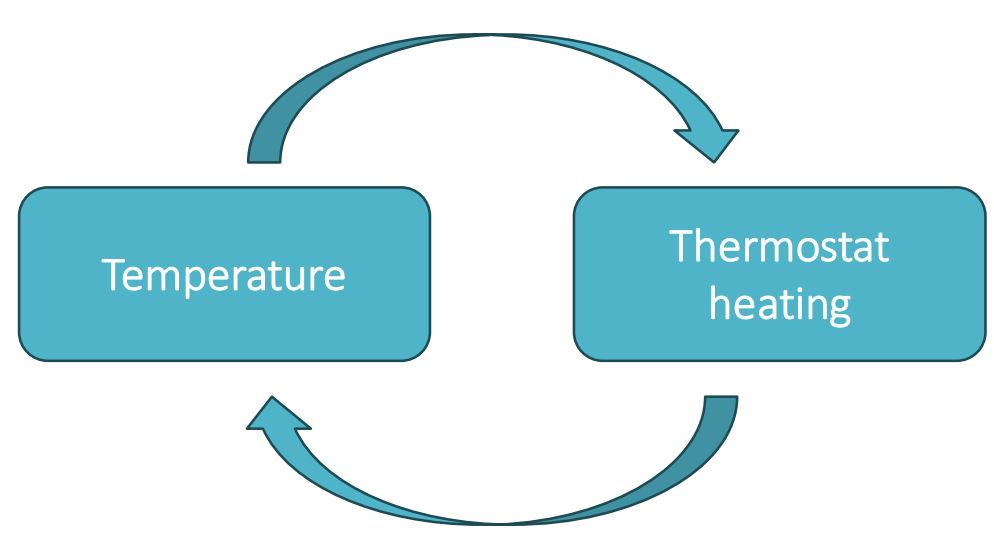

22

How can process characteristics create a potential for tipping through feedbacks?

Process	Drivers	Direct Impacts	Indirect Impacts
Economic growth in natural resource industry (e.g., fisheries)	- Exogenous demand for product<br>- Resource availability	- Financial returns<br>- Job availability<br>- Resource availability	- Perceptions of resource health and availability
Migration in and out of community	- Housing costs, availability<br>- Perceptions of community vitality, desirability	- Population<br>- Housing costs, availability	- Tax base<br>- Perceptions of community vitality, desirability

23

Cascading linkages

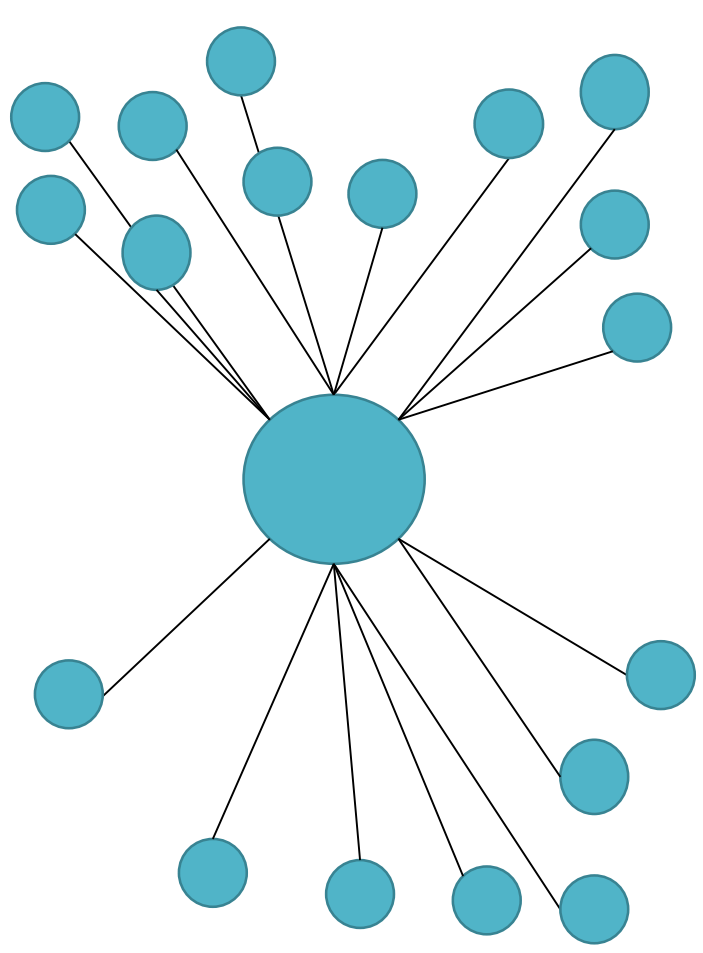

- Many connections between people, business, and physical resources of a community
- Certain network structures can lead to greater impacts:
  - Hubs (central nodes that connect with many other elements)
  - Paths (chain of elements that impact each other)
- Example: loss of a key industry or business can ripple out to many connected businesses

24

Cascading Linkages

Wolf population

Deer population

Vegetation in valleys

Erosion rates

River morphology

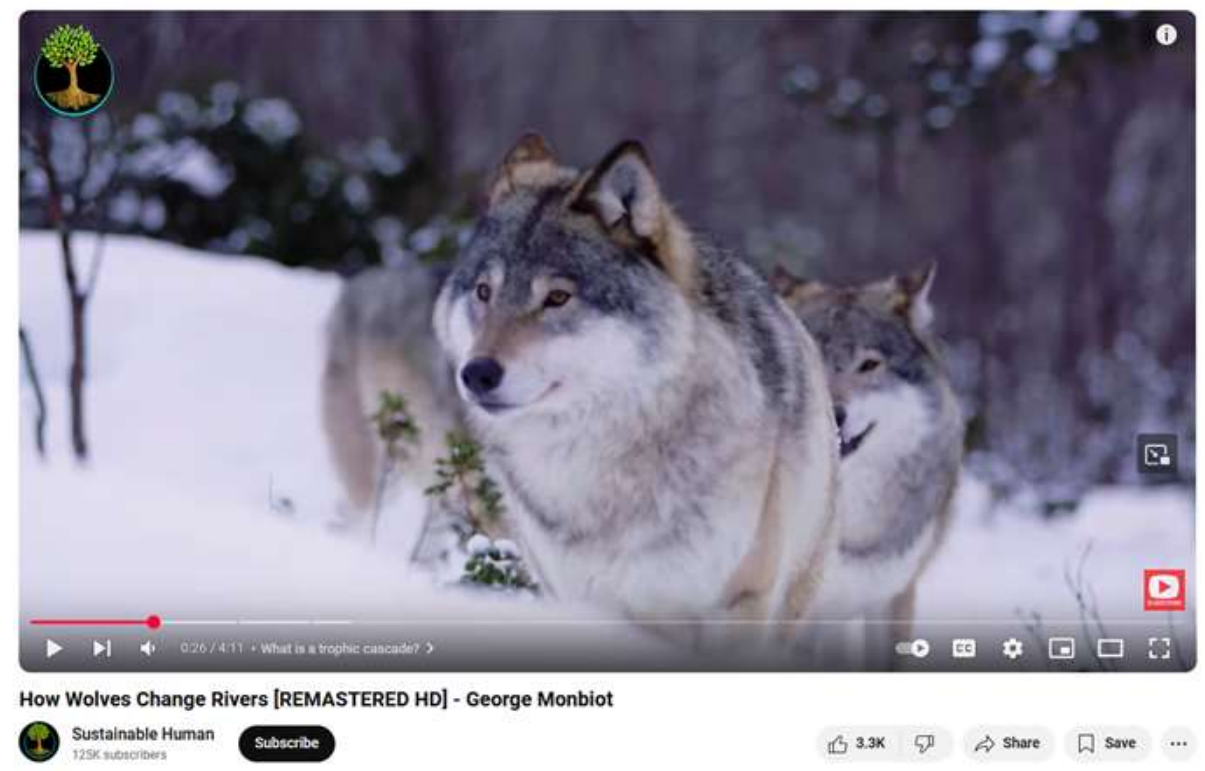

25

Nonlinear Relationships

- Gradual change in physical conditions lead to large jump in impacts

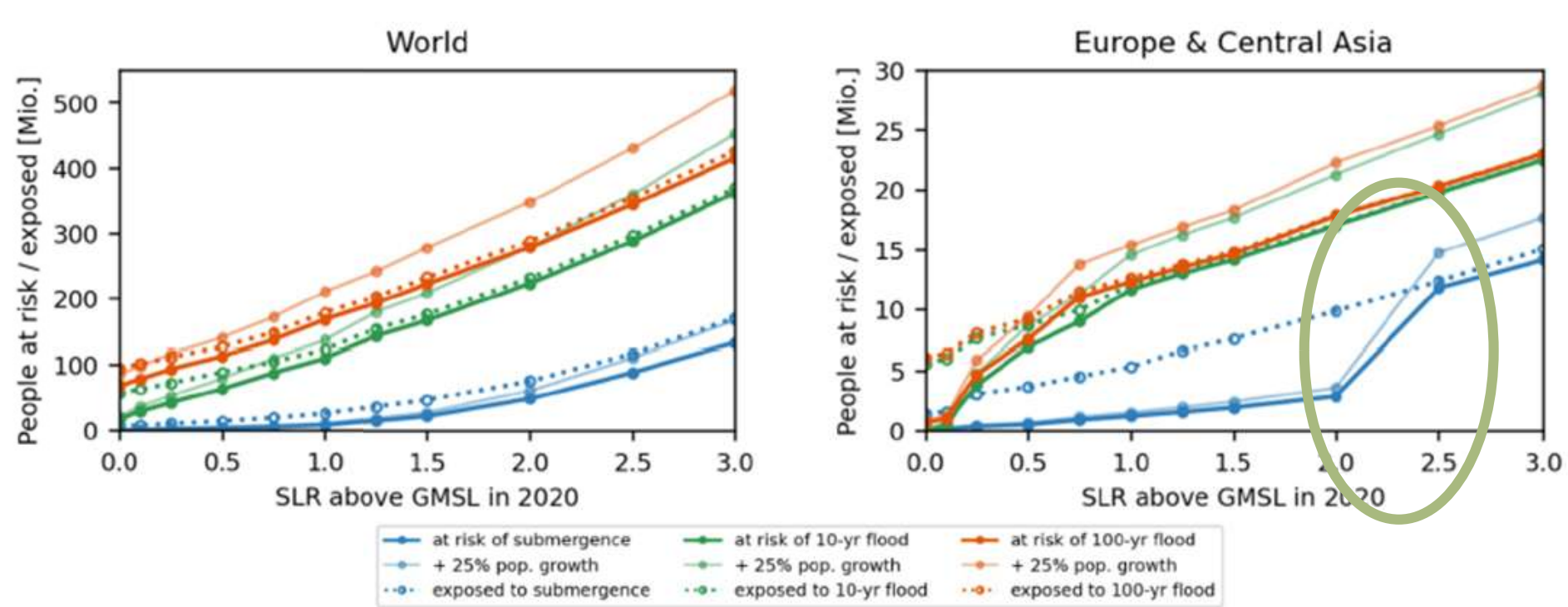

Haasnoot et al., 2021. *Climate Risk Management.* 34 (100355)

26

Nonlinear Relationships

- Can arise from feedbacks and linkages:
  - Investment in public awareness programs (people reached increases if word spreads organically through linkages)
- Can arise independently
  - Coastal hyposemetry (elevation vs. area curve)
  - Threshold effects (e.g., species presence/absence after water pollution)
  - Business profit and number of employees
- Can take different forms, including thresholds

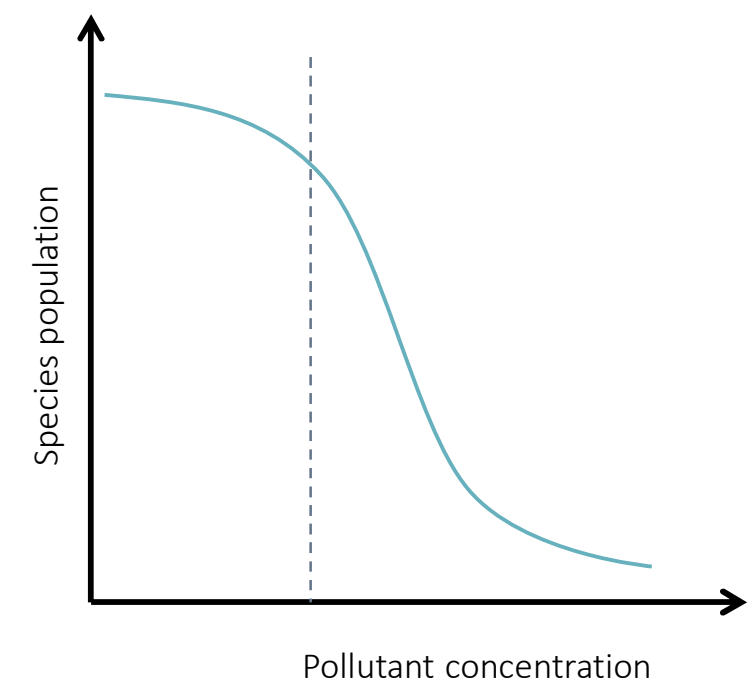

27

Identifying Potential Tipping Points

Small group discussions: 12:50 – 13:40

Review systems inventory components from morning.

1. Feedback processes: Processes where drivers and results overlap
2. Linkages: Identifying hubs and pathways
3. Nonlinear relationships: linkages between elements that are likely to exhibit threshold behavior

Keep in mind:

- Tipping points may not fit cleanly into a single category, and that is ok.

- An exhaustive list of potential tipping points is not the goal. A thorough description of a small number is better than a simple list of many.

28

Ranking Tipping Points

- Have some coffee
- Scan QR Code to access tipping point ranking

menti.com
Code: 2195 8846

29

Data needs and research to inform tipping points

Individual Reflection:
14:30 – 14:35
Group Discussion:
14:35 – 14:45

For the tipping points you think are most interesting:

- What are the key questions or research priorities that would help us better understand this tipping point?
- What methods are available to gather or generate this data? What types of expertise are necessary?
- What partners (academic or non) are needed to address this question?

30

Closing Comments

- Thank you!!
- Future steps include manuscript and proposal development that build upon discussions
- All workshop materials and outputs will remain available online for you to view as interested

Links to closing survey:

Menti.com
Code: 7603 4042

31
